# Metabolic Reprogramming of Tumor-Associated Macrophages Using Glutamine Antagonist JHU083 Drives Tumor Immunity in Myeloid-Rich Prostate and Bladder Cancers

**DOI:** 10.1158/2326-6066.CIR-23-1105

**Published:** 2024-04-26

**Authors:** Monali Praharaj, Fan Shen, Alex J. Lee, Liang Zhao, Thomas R. Nirschl, Debebe Theodros, Alok K. Singh, Xiaoxu Wang, Kenneth M. Adusei, Kara A. Lombardo, Raekwon A. Williams, Laura A. Sena, Elizabeth A. Thompson, Ada Tam, Srinivasan Yegnasubramanian, Edward J. Pearce, Robert D. Leone, Jesse Alt, Rana Rais, Barbara S. Slusher, Drew M. Pardoll, Jonathan D. Powell, Jelani C. Zarif

**Affiliations:** 1 Pathobiology Graduate Program, Johns Hopkins University School of Medicine, Baltimore, Maryland.; 2 Bloomberg∼Kimmel Institute, Johns Hopkins University School of Medicine, Baltimore, Maryland.; 3 Department of Oncology, Johns Hopkins University School of Medicine, Baltimore, Maryland.; 4 The Sidney Kimmel Comprehensive Cancer Center, Johns Hopkins University School of Medicine, Baltimore, Maryland.; 5 Graduate Program in Immunology, Johns Hopkins University School of Medicine, Baltimore, Maryland.; 6 Medical Scientist Training Program, Johns Hopkins University School of Medicine, Baltimore, Maryland.; 7 Department of Medicine, Center for Tuberculosis Research, School of Medicine, Johns Hopkins University, Baltimore, Maryland.; 8 Graduate Program in Biomedical Engineering, Johns Hopkins University School of Medicine, Baltimore, Maryland.; 9 Graduate Program in Cellular and Molecular Medicine, Johns Hopkins University School of Medicine, Baltimore, Maryland.; 10 Department of Neurology, Johns Hopkins School of Medicine, Baltimore, Maryland.; 11 Johns Hopkins Drug Discovery, Johns Hopkins School of Medicine, Baltimore, Maryland.

## Abstract

Glutamine metabolism in tumor microenvironments critically regulates antitumor immunity. Using the glutamine-antagonist prodrug JHU083, we report potent tumor growth inhibition in urologic tumors by JHU083-reprogrammed tumor-associated macrophages (TAMs) and tumor-infiltrating monocytes. We show JHU083-mediated glutamine antagonism in tumor microenvironments induced by TNF, proinflammatory, and mTORC1 signaling in intratumoral TAM clusters. JHU083-reprogrammed TAMs also exhibited increased tumor cell phagocytosis and diminished proangiogenic capacities. *In vivo* inhibition of TAM glutamine consumption resulted in increased glycolysis, a broken tricarboxylic acid (TCA) cycle, and purine metabolism disruption. Although the antitumor effect of glutamine antagonism on tumor-infiltrating T cells was moderate, JHU083 promoted a stem cell–like phenotype in CD8^+^ T cells and decreased the abundance of regulatory T cells. Finally, JHU083 caused a global shutdown in glutamine-utilizing metabolic pathways in tumor cells, leading to reduced HIF-1α, c-MYC phosphorylation, and induction of tumor cell apoptosis, all key antitumor features. Altogether, our findings demonstrate that targeting glutamine with JHU083 led to suppressed tumor growth as well as reprogramming of immunosuppressive TAMs within prostate and bladder tumors that promoted antitumor immune responses. JHU083 can offer an effective therapeutic benefit for tumor types that are enriched in immunosuppressive TAMs.

## Teaser

The authors show in myeloid-rich urologic tumors that glutamine antagonism in TAMs results in functional alterations including increased tumor-cell phagocytosis, inflammatory signaling, immune-proliferative pathways, and glycolysis. This promotes antitumor immunity, suggesting new therapeutic approaches for these immunotherapy-unresponsive tumors.

## Introduction

Tumor-associated macrophages (TAM) are critical, heterogeneous immune cells that support tumor growth in the tumor microenvironment (TME). TAMs most often facilitate tumor progression by promoting metastasis, dampening antitumor adaptive immunity, inducing immune suppression via secreted protumoral metabolites, and promoting tissue repair in solid neoplasms (1, 2). Both prostate and bladder cancers boast a high abundance of immunosuppressive TAMs, which are associated with worse prognosis and are known to facilitate chemotherapeutic resistance (3–5), making them a translationally important intratumoral immune compartment.

Unfortunately, current immune checkpoint blockade (ICB) therapies have been minimally successful for treating urological cancers, especially metastatic castration-resistant prostate cancers (mCRPC) (6–8), which have a ∼30% 5-year survival rate and kill ∼34,000 men in the United States annually (9). More effective novel myeloid-targeting immunotherapies are urgently needed, especially for late-stage disease, which features a myeloid-rich and poor lymphocyte-infiltrating TME (10, 11). Recently, progress on such therapies has been made in the form of an array of preclinical combination therapies targeting myeloid cell recruitment (CSF1R and CCR2), phagocytosis (CD47 and SIRPα), activation (TLRs and CD40), reprogramming/polarization (PI3Kγ and Stat3), and metabolism (IDO inhibitors and selective class IIa HDAC inhibitors), moving forward to evaluation in phase I/II trials in solid primary malignancies (2, 12, 13). However, completed phase II clinical trials for CSF1R inhibition have shown no durable responses (3).

TAMs demonstrate rapid adaptability into diverse phenotypic, metabolic, and functional states in the TME in response to environmental cues (2). Conventionally, whereas M1-like macrophages are increasingly glycolytic, coupled with excess lactate secretion and increased NADPH, lipid, and nucleotide biosynthesis, M2-like macrophages utilize oxidative phosphorylation (OXPHOS) and glutaminolysis to meet bioenergetic demands (2, 14). TAMs are largely polarized toward an immunosuppressive M2 phenotype intratumorally; however, this overly simplistic description of metabolic status does not fit well with TAM behavior because of their heterogeneity and the plasticity they exhibit (15). Glutamine plays a well-established role in macrophage activation, and both human and murine TAMs exhibit elevated levels of glutamine transporters and metabolic enzymes and exhibit increased glutamine consumption (16–19). The key glutamine-synthesizing enzyme, glutamate-ammonia ligase (GLUL), is upregulated in most human cancers and in M2-like macrophages (18, 20). Pharmacological inhibition of GLUL skews M2-polarized macrophages towards the M1-like phenotype, characterized by reduced intracellular glutamine. In addition, genetic deletion of GLUL inhibits tumor metastasis in a process characterized by increased tumor vessel pruning and inhibition of seeding of metastatic lung tumors (19), suggesting a critical role for glutamine metabolism in immune suppression and tumor metastasis. Moreover, Xu and colleagues demonstrated the therapeutic benefits of targeting glutaminase 1 (GLS1) in radioresistant prostate cancer cells (21).

The broadly active glutamine antagonist 6-diazo-5-oxo-L-norleucine (DON) has shown potent tumor toxicity and tremendous therapeutic efficacy across several indications (22, 23). However, severe dose-limiting gastrointestinal toxicities associated with DON have led to the termination of studies for its clinical development (22). Importantly, DON-induced glutamine antagonism causes broad inhibition of many glutamine-utilizing enzymes such as glutaminase and multiple glutamine amidotransferases involved in nucleotide synthesis, amino acid synthesis, hexosamine production, and glutamine transportation (22). As such, DON offers a unique, broad, yet specific glutamine antagonistic approach, which likely reduces the risk of single mutations or other resistance mechanisms developing during DON treatment.

We wanted to harness the impressive antitumor effects of DON while addressing its observed toxicities. Therefore, we used a well-tolerated DON prodrug, JHU083, which activates within the TME and thus limits systemic toxicities (24). JHU083 has been shown to cause significant tumor growth inhibition (TGI), attenuate metastatic progression, and improve animal survival in a variety of syngeneic murine models of melanoma, colon cancer, lymphoma, immunotherapy-resistant triple-negative breast cancer, and glioma (25). Metabolic targeting of the TME with JHU083 has shown that the compound has a tumoricidal effect on tumor cells while potentiating a CD8^+^ T cell response due to the metabolic plasticity of these cells. In a landmark study, Oh and colleagues (26) demonstrated in a murine 4T1 breast cancer model that JHU083 decreased the recruitment of immunosuppressive myeloid-derived suppressor cells (MDSC), increased immunogenic cell death, and promoted the repolarization of MDSCs to proinflammatory macrophages within the TME, suggesting that MDSCs are prominent cell types affected by JHU083 (26). However, we lack a detailed understanding of the metabolic and functional aspects of reprogramming intratumoral TAMs upon glutamine antagonism (27).

Here, we provide evidence of increased glutamine metabolism in TAMs in human mCRPC samples taken from bone metastases and show that inhibition of glutamine utilization after JHU083 treatment was robust in three murine syngeneic, myeloid-rich urologic tumor models. We show that JHU083 had a potent and direct antitumor effect and reprogramed the TME by exploiting the differential metabolic plasticity between tumor cells, macrophages, and T cells. Additionally, we report that JHU083-mediated restoration of antitumor immunity was largely due to reprogramming of myeloid cells (TAMs and tumor-infiltrating monocytes (TIMs) such that they showed increased phagocytosis, proliferation, inflammatory signaling, blockade of purine metabolism, and glycolysis and reduced α-ketoglutarate-driven tumor-toxic proinflammatory signaling.

## Materials and Methods

### Animals

Experimental protocols involving live animals were performed in accordance with the protocols approved by the Institutional Animal Care and Use Committee at the Johns Hopkins University School of Medicine. Male and female C57BL/6J (000664) mice, ages 6 to 8 weeks, were purchased from The Jackson Laboratory. Animals were housed under standard conditions (68°F–76°F, 30%–70% relative humidity, 12:12 light–dark cycle) with free access to standard chow and water. The general behavior and appearance of the animals were monitored daily by veterinary specialists.

### Tumor models and cell lines

MB49, a mouse urothelial carcinoma cell line (SC148) derived from an adult C57BL/6J mouse by exposure of a primary bladder epithelial cell explant to 7,12-dimethylbenz [a]anthracene (DMBA) for 24 hours followed by a long-term culture, was purchased from Sigma in 2019. PC3 cells (prostate adenocarcinoma cells, CRL1435) were purchased from ATCC in 2018. Human umbilical vein endothelial cell 2 (HUVEC2, 354151) were purchased from Corning Life Science in 2022. B6CaP tumor cells were a gift from Dr. Brian Simons (Baylor College of Medicine). RM1 (CRL3310), a mouse prostate carcinoma cell line of fibroblast-like morphology, was purchased from ATCC in 2023. MB49-luciferase RFP cells (SC065-R) were purchased from GenTarget in 2021. RM1-Luc-RFP cells were generated using viral transduction of RM1 cells with Luciferase (firefly)-2A-RFP (EF1a, Puro) (LVP440-PBS; GenTarget Inc.) at a multiplicity of infection of 1:10. Short tandem repeats and mycoplasma testing were performed on each human cell line at the start of experimentation. MB49, RM1, MB49-RFP, and RM1-Luc-RFP cells were cultured in DMEM (11965092, Gibco) supplemented with 10% FBS (100-106, GeminiBio) and 1% penicillin/streptomycin (15140122, Gibco) at 37°C with 5% CO_2_. PC3 cells were maintained in F12K medium (21127022, Gibco) supplemented with 10% FBS and 1% penicillin/streptomycin at 37°C with 5% CO_2_. HUVEC2 cells were maintained in human large vessel endothelial cell basal medium (M200PRF500, Gibco) supplemented with a low serum growth supplement kit (S003K, Gibco) at 37°C with 5% CO_2_ and passaged no more than five times. Cells were harvested following trypsinization, and cell viability was confirmed using trypan blue dye. For syngeneic heterotopic MB49 urothelial tumor and RM1 prostate cancer development, live MB49 cells (5.0 × 10^4^ cells per 100 μL of 1× PBS per mouse) were implanted at the right flank of C57BL/6J female mice. The RM1 prostate cancer model was established by injecting 5.0 × 10^4^ cells in 100 μL of 1× PBS at the right flank of C57BL/6J male mice. To develop the syngeneic heterotopic prostate carcinoma tumors B6CaP, CD45^−^ cells were thawed, washed with 1× PBS, and implanted subcutaneously (5.0 × 10^5^ cells per 100 μL 1× PBS per mouse) on the right flank of C57BL/6J male mice for passaging of the cells in mice. Once the tumors reached 1,000 mm^3^, they were harvested and implanted after enrichment for CD45^−^ cells using CD45 microbeads (130-052-301, Miltenyi Biotec), as per the manufacturer’s protocol. Briefly, the cell suspension was incubated with CD45 microbeads and passed through the MACS column (130-042-401, Miltenyi Biotec), in which the flow-through that contained CD45^−^ cells was collected. Tumor growth was monitored every second day to observe the increase in tumor burden at the time of treatment initiation. Tumors were measured using an electronic caliper, and tumor volume was calculated using the following equation: tumor volume = length × width × height × 0.5.

### DON and JHU083 treatment

DON (D2141) was purchased from Sigma-Aldrich. JHU083 (ethyl 2-(2-amino-4-methylpentanamido)-DON) was synthesized as previously described and was provided by Dr. Barbara Slusher (Johns Hopkins University). Briefly, JHU083 was administered orally (p.o.) at a dose of 1 mg/kg DON molar equivalent in 1× sterile PBS. Once palpable, tumor-bearing C57BL/6J mice were orally treated with JHU083 or vehicle for 5 to 9 days daily and then at a lower dose of 0.3 mg/kg DON equivalent. For all drug administrations, care was taken to handle the animals gently to minimize stress.

### *In vivo* drug treatment and cell-specific depletion

Mice bearing palpable tumors (100–500 mm^3^) were treated with vehicle, referred to as control (1× sterile PBS), or with JHU083 (1 mg/kg DON equivalent) daily for 7 or 9 days. Then, a reduced dosage of 0.3 mg/kg DON equivalent was administered daily until the vehicle control tumors reached a maximum tumor volume of 2,000 mm^3^. For CD4^+^ T cell depletion, mice were injected intraperitoneally (i.p.) with 200 μg of anti-CD4 (InVivoPlus GK1.5, BP0003-1, Bio X Cell) or isotype control (InVivoPlus rat IgG2b isotype control, antikeyhole limpet hemocyanin, LTF2, BP0090, Bio X Cell) in 100 μL of 1× PBS on day 3 prior to tumor inoculation and then once every week until the end of the experiment. For CD8^+^ T cell depletion, the mice were injected with 200 μg anti-CD8β (InVivoMAb Lyt 3.2, 53-5.8, BE0223, Bio X Cell) or isotype control (InVivoMAb rat IgG1 isotype control, antihorseradish peroxidase, clone HRPN, BE0088, Bio X Cell) in 100 μL of 1× PBS on day three prior to tumor inoculation. The anti-CD4 and anti-CD8 dosing schedules were optimized in nontumor-bearing C57BL/6J mice, and the spleen was tested by flow cytometry to quantify depletion. For studies combining anti-PD1 therapy, MB49 tumor-bearing mice were treated intraperitoneally with either 250 μg anti-PD1 (InVivoPlus RMP1-14, #BP0146, Bio X Cell) or JHU083 (in a similar dosing scheme as mentioned), or both or isotype control (InVivoPlus rat IgG2a isotype control, anti-trinitrophenol, 2A3, BP0089, Bio X Cell) on every third day after MB49 cell implantation. Two biologically separate experiments were performed to confirm the phenotype, with *n* = 3–10 in each group within one experiment. For optimization of macrophage depletion, anti-CSF1R (InVivoMAb AFS98, BE0213, Bio X Cell) was tested in nontumor-bearing C57BL/6J mice, and the spleen was tested by flow cytometry to quantify macrophage depletion on day 3 after dosing with 300 μg.

### Macrophage depletion with clodronate

Six-week-old female C57BL/6J mice were pretreated with 200 μL of either clodronate encapsulated in liposomes or the control liposome Standard Macrophage Depletion Kit (Clodrosome^®^ + Encapsome^®^, CLD-8901, Encapsula NanoSciences) via retro-orbital injection. After 48 hours, 5.0 × 10^4^ MB49 cells were implanted in the right flank subcutaneously. Mice were dosed with clodronate, or liposome control, every 4 days throughout the experiment. Once tumors became palpable, mice were randomized into different groups. In groups that received JHU083, dosage was given as described previously (see “DON and JHU083 treatment”). Tumor volume was measured as described previously (see “Tumor models and cell lines”).

### Adoptive transfer experiments

Following the development of palpable MB49 tumors (100–500 mm^3^), tumor-bearing C57BL/6J mice were orally given JHU083 daily for 5 days and then a reduced dose of 0.3 mg/kg equivalent of DON until the experimental endpoint was met. TAMs (live CD45^+^CD3^−^Ly6G^−^CD11b^+^F4/80^+^) were isolated from the donor mice (vehicle control or JHU083-treated; see “Tumor digestion, flow cytometry, and sorting”) and then admixed at a 1:1 ratio with MB49 tumor cells, and 60,000 cells in total were injected subcutaneously into recipient syngeneic C57BL/6J female mice for tumor development. This was followed by tumor volume measurements after palpable tumors developed. Similarly, for TIM adoptive transfers (AT), TIMs (live CD45^+^CD3^−^CD11b^+^Ly6G^−^Ly6C^high^) were separately sorted from both JHU083- and vehicle (1× sterile PBS)-treated mice. Sorted TIMs were then mixed with MB49 in a 1:1 ratio and injected subcutaneously into recipient syngeneic C57BL/6J female mice. Two biologically separate experiments were performed to confirm the phenotype, with *n* = 5–10 in each group within one experiment.

### Tumor digestion, flow cytometry, and fluorescence-activated cell sorting

Tumors were surgically resected, mechanically minced, and digested using Miltenyi’s Mouse Tumor Dissociation Kit (130-096-730), according to the manufacturer’s protocol, using a gentleMACS Octo Dissociator (130-096-427). After tumor digestion, the cells were filtered through a 100-μm cell strainer (TC70-MT2, Stellar Scientific). For flow cytometry, single-cell suspensions were washed with 1× PBS and then incubated with ACK lysis buffer (118-156-721, Quality Biologicals). For FACS, cells were washed, and tumor-infiltrating CD45^+^ cells were enriched using the mouse CD45 isolation kit (130-052-301, Miltenyi), according to the manufacturer’s protocol. After staining, TAMs or TIMs were sorted using a BD FACSAria Fusion. Single-cell suspensions were stained with antibodies after viability staining and Fc Receptor blocking (553142; BD Biosciences). Supplementary Table S1 contains the list of mouse and human antibodies used in this study. Staining was performed according to the manufacturer’s instructions. For intracellular staining, the eBioscience Foxp3/Transcription Factor Staining Buffer Set (00-5523-00, Thermo Fisher Scientific) was used according to the manufacturer’s protocol. Cells were washed and immunophenotyped using a BD FACSCelesta, BD FACS Symphony, or Cytek Aurora, and data were analyzed using FlowJo (version 9 or 10). Bulk RNA sequencing (RNA-seq) was performed using sorted TAMs (live CD45^+^CD3^−^Ly6G^−^CD11b^+^F4/80^+^) from vehicle or JHU083-treated B6CaP tumors.

### RNA preparation, bulk RNA-seq, and data analysis

Total RNA was isolated from vehicle (*n* = 3) and JHU083-treated (*n* = 3) tumors using TRIzol (15596026, Thermo Fisher Scientific) according to the manufacturer’s protocol. For RNA-seq, RNA samples were converted to double-stranded cDNA using the Ovation RNA-Seq System v2.0 kit (Tecan), which utilizes a proprietary strand displacement technology for linear amplification of mRNA without rRNA/tRNA depletion as per the manufacturer’s recommendations. This approach does not retain strand-specific information. The quality and quantity of the resulting cDNA were monitored using the Bioanalyzer High Sensitivity kit (Agilent), which yielded a characteristic smear of cDNA molecules ranging in size from 500 to 2,000 nucleotides in length. After shearing 500 ng of cDNA to an average size of 250 nucleotides with the Covaris S4 (Covaris Inc.), the mRNA libraries were constructed using the TruSeq Nano kit (Illumina) according to the manufacturer’s instructions. The mRNA libraries were sequenced on an Illumina NovaSeq 6000 instrument using 150-bp paired-end dual-indexed reads and 1% of PhiX control. The reads were aligned to the genome build (mm39). rsem-1.3.0 was used for alignment and for generating gene expression levels. The “rsem-calculate-expression” module was used with the following options: -star, -calc-ci, -star-output-genome-bam, -forward-prob 0.5. Differential expression analysis and statistical testing were performed using DESeq2 software. The identified list of significantly differentially expressed genes (DEG) was then enriched for their biological functions to explore and evaluate their involvement in critical biological processes in the context of the study. We employed gene set enrichment analysis (GSEA) using the R statistical tool to screen for statistically significant, cumulative changes in groups of genes in the context of pathway analysis.

### Single-cell RNA-seq and data analysis

Sorted CD45^+^ and CD45^−^ cells from B6CaP tumors were used for single-cell RNA-seq (scRNA-seq). Briefly, cell counts and viability were determined using Cell Countess 3 with trypan blue. A maximum volume of 86.4 μL/sample was used for processing to target up to 20,000 cells. Cells were combined with Reverse Transcription (RT)reagents and loaded onto 10× Next GEM Chip M along with 3′-HT gel beads. The NextGEM protocol was run on the 10× Chromium X to create GEMs (gel beads in emulsion), composed of a single cell, gel beads with a unique barcode and UMI primer, and RT reagents. Then, 180 μL of emulsion is retrieved from the chip, split into two wells, and incubated (45 minutes at 53°C, 5 minutes at 85°C, cooled to 4°C), generating barcoded cDNA from each cell. The GEMs were broken using a Recovery Agent, and cDNA was cleaned following the manufacturer’s instructions using MyOne SILANE beads. cDNA was amplified for 11 cycles (3 minutes at 98°C, 11 cycles: 15 seconds at 98°C, 20 seconds at 63°C, 1 minute at 72°C; 1 minute at 72°C, cool to 4°C). The samples were cleaned using 0.6× SPRIselect beads. Quality Control was completed using a Qubit and Bioanalyzer to determine the size and concentration. Amplified cDNA (10 µL) was added to the prep library. Fragmentation, end repair, and A-tailing were completed (5 minutes at 32°C, 30 minutes at 65°C, cooled to 4°C), and samples were cleaned up using double-sided size selection (0.6×, 0.8×) with SPRIselect beads. Adaptor ligation (15 minutes at 20°C, cooled to 4°C), 0.8× cleanup, and amplification were performed with PCR using unique i7 index sequences. Libraries underwent a final cleanup using double-sided size selection (0.6×, 0.8×) with SPRIselect beads. Library QC was performed using the Qubit, Bioanalyzer, and KAPA library quantification qPCR kits. Libraries were sequenced on the Illumina NovaSeq 6000 using v1.5 kits, targeting 50,000 reads/cell at read lengths of 28 (R1), 8 (i7), and 91 (R2). Demultiplexing and FASTQ generation were completed using the Illumina BaseSpace software. FASTQ files were processed using Cell Ranger (version 7.0.0.) using mm10 as the reference genome, resulting in a total of 101,670 cells. After filtering out low-quality cells, red blood cells, and doublets, 84,643 cells remained for downstream analyses.

Cells with more than 10% mitochondrial gene content or less than 250 detected mitochondrial genes were filtered out and excluded from downstream analysis. Doublets were removed using the DoubletFinder pipeline (28) by first calculating an optimal pK value for each sample with the paramSweep_v3 () function, with an expected per-sample doublet rate of 6%. Log-normalized counts were used to automatically annotate individual cells using the SingleR algorithm with coarse labels from celldex::ImmGenData as a reference (29). Data were then processed with the Seurat pipeline (30) using the top 3,000 variable genes and regressing out percentage mt and the difference between G2/M and S phase scores determined by CellCycleScoring to retain separation between cycling and noncycling cells. Louvain clustering and Uniform Manifold Approximation and Projection (UMAP) for Dimension Reduction were performed on the top 30 principal components. To further investigate specific immune cell subsets, we selected clusters based on SingleR annotations of the full CD45^+^ fraction: either macrophages/monocytes or T cells/NK cells. Reciprocal Principal Component Analysis integration was performed on each subset to account for sample-to-sample heterogeneity, followed by the Seurat pipeline as described above. Clusters at the subset level were manually annotated based on basic marker genes for monocytes (*Itgam*, *Ly6c2*, and *Ccr2*), macrophages (*Adgre1* and *Mrc1*), T cells (*Cd3d*, *Cd3g*, *Cd3e*, *Cd4*, and *Cd8a*), Tgd (*Trdc* and *Tcrg-C1*), and NK cells (*Ncr1*, *Klrb1c*, *Prf1*, and *Gzmb*). The IFN_TAM and Glycolytic_TAM populations were labeled based on their expression of interferon-responsive genes (*Ifit1*, *Ifit2*, *Ifit3*, *Isg15*, *Iigp1*, and *Ifi47*) and glycolysis-related genes (*Slc2a1*, *Pfkp*, and *Aldoa*), respectively. Differential expression analysis between JHU083-treated and nontreated controls was performed using the Wilcoxon ranked-sum test with the wilcoxauc function from the presto package (31). Phagocytosis cell scores were calculated based on the following phagocytosis-related genes: *Rac1*, *Dock2*, *Rhoa*, *Nckap1l*, *Wasf2*, *Abi1*, *Cyfip1*, *Brk1*, *Actr2*, *Actr3*, *Arpc2*, *Arpc3*, *Arpc4*, *Rraga*, *Lamtor2*, *Lamtor3 Lamtor4*, *Nprl2*, *Nhlrc2*, *Tm2d1*, *Tm2d2*, *Tm2d3*, *Itgb2*, *Tln1, Fermt3*, *Pp2a*, *Mapk*, *Pkc*, *Plek*, *Rab1a*, *Rab2a*, *Rab5a*, *Rab10*, *Rab11a*, *Rab14*, *Rab20*, *Rab22a*, *Rab32*, *Rab34*, *Rab7b*, and *Myo1e* (32, 33).

To identify which macrophage cluster from the early time point (single-cell experiment) matched the transcriptional changes seen in macrophages at the later point (bulk experiment), we performed GSEA on each cluster between JHU083-treated and control samples. The lists of significantly upregulated and downregulated genes identified in the macrophage-sorted bulk RNA-seq experiment (late time point) were used as input as the respective “up” or “down” gene sets in the GSEA. A score was calculated to rank each macrophage cluster based on the enrichment of both up- and down-regulated gene sets by summing the *P*-value-weighted absolute values of each normalized enrichment score:score = |NESup| × −log_10_ (adj.pup) + |NESdown| × −log_10_ (adj.pdown). For RNA velocity analysis, bam files generated for each sample from the Cell Ranger pipeline were prepared using samtools sort (34), followed by velocyto run10× (35) with the mm10 repeat mask downloaded from the UCSC table browser and the mm10 genome annotation file provided by the Cell Ranger. Loom files containing spliced and unspliced counts from velocyto were filtered to include only cells that passed previous quality checks in the macrophage/monocyte subset, and scVelo was used to calculate splicing kinetics through dynamical modeling (36).

### Analysis of publicly available data

The previously published and publicly available scRNA-seq human prostate cancer bone metastasis data published by Kfoury and colleagues (41) were downloaded as an RData object provided through the author’s website: https://pklab.org/bonemet. This dataset includes scRNA-seq data on bone marrow samples from seven patients undergoing hip replacement surgery and patient-matched metastatic tumors, involved bone marrow, and distal bone marrow from nine patients with metastatic castration-resistant prostate cancer. Gene expression count matrices and cell type annotations can be accessed from the Gene Expression Omnibus (GEO) with the accession number GEO GSE143791. The data were then converted to a Seurat in the R programming language for further analysis by extracting the expression matrix, cluster assignments, and t-distributed Stochastic Neighbor Embedding embeddings from the Conos object (30, 37).

### Targeted metabolomics and pathway analysis

Metabolites were extracted from flash-frozen whole tumors normalized by dry weights for each individual tumor (B6CaP or MB49) or sorted TAMs (from B6CaP tumors) with 80% methanol (80% methanol: 20% water: v/v). Supernatants were isolated after centrifugation at high speed (15,000 × *g*) for 10 minutes, dried under nitrogen gas, and stored at −80°C for LC-MS analysis. For analysis, the dried metabolite extracts were dissolved in 50% acetonitrile (ACN) solution, and metabolome profiling was performed on an Agilent LC-MS/MS system [timsTOF Pro II mass spectrometer (Bruker Daltonics) equipped with an electrospray ionization (ESI) source]. The LC-MS/MS parameters used were as previously described (27). Relative metabolite abundance was plotted and normalized (per mg of tumor tissue or cell count) using MetaboAnalyst 5.0.

### Absolute quantification of intratumoral metabolites

LC-MS–based absolute quantifications of glutamine, glutamate, and glucose were performed using a validated method as previously described (38). Briefly, glutamine and glutamate were extracted via a one-step protein precipitation. Five microliters of methanol containing 10 μmol/L deuterated glutamate, glutamate, and glucose (internal standard) was added per milligram of tissue. Samples were centrifuged at 16,000 × *g* for 5 minutes at 4°C. A standard concentration curve of glutamate and glutamine was prepared (0.1–10,000 μmol/g). The samples were analyzed using an Agilent 1290 UPLC coupled to an Agilent 6520 quadrupole time-of-flight mass spectrometer. Samples (2 µL) were injected and separated on a Waters Acquity UPLC BEH Amide 1.7 µm 2.1 × 100 mm Hydrophilic Interaction Liquid Chromatography (HILC) column with a flow rate of 0.3 mL/minute. The mobile phase consisted of A (water + 0.1% formic acid) and B (ACN + 0.1% formic acid). The mass spectrometer, equipped with a dual ESI ionization source, was run in positive and negative ion modes for glutamine and glutamate and then for glucose analysis. Data were acquired and quantified using MassHunter software. The absolute quantification of formylglycinamide ribonucleotide (FGAR) was performed as previously described (39) with minor modifications. Briefly, FGAR was extracted from tumors using the protein precipitation method. Five microliters of methanol containing 10 μmol/L deuterated N-acetylaspartic acid (internal standard) was added per milligram of tissue. The tissue samples were homogenized and centrifuged (16,000 × *g* for 5 minutes). For quantification, the supernatants (2 μL) were injected and separated on an UltiMate 3000 UHPLC coupled to a Q Exactive Focus orbitrap mass spectrometer (Thermo Fisher Scientific Inc.). Samples were separated on an Agilent Eclipse Plus C18 RRHD (1.8 μm) 2.1 × 100 mm column. The mobile phase consisted of 8 mmol/L dimethylhexylamine + 0.005% formic acid in water, pH 9 (A), and 8 mmol/L dimethylhexylamine in ACN (B). The separation was achieved at a flow rate of 0.4 mL/minute using a gradient run. Quantification was performed in full MS negative mode. Data were acquired and quantified using Xcalibur software.

### *In vivo* glucose tracing in TAMs

A 20% (w/v) solution of [U-^13^C] glucose (CLM-1396-PK, Cambridge Isotope Labs) was injected intravenously thrice at 15-minute intervals in restrained B6CaP tumor-bearing mice without anesthesia (27). The tumors were harvested 45 minutes after the first injection. Rapid tumor digestion for 10 minutes was performed in 1× PBS following passage through cell strainers and ammonium-chloride-potassium (ACK) lysing buffer, as mentioned previously. Tumor-infiltrating CD45^+^ cells were enriched for 10 minutes using a mouse CD45 isolation kit (130-052-301, Miltenyi), followed by fast staining in a pre-made antibody cocktail for FACS. TAMs were sorted using a BD FACSAria Fusion, and the protocol for polar metabolite isolation was followed as previously described (see “Targeted metabolomics and pathway analysis”). The cold chain at 4°C was maintained throughout the protocol and cells, except for digestion.

### DON treatment of human monocyte-derived macrophages

Briefly, CD14^+^ monocytes were isolated from consented healthy donor leukopaks from Johns Hopkins Hospital using the Human CD14 Positive Selection Kit (17858, STEMCELL Technology), differentiated, and polarized into M1-like or M2-like macrophages as previously reported (40). Briefly, CD14^+^ monocytes were seeded at a cell density of 2.0 to 3.0 × 10^5^ cells/mL in RPMI 1640 medium (11875093, Gibco) supplemented with 10% heat-inactivated FBS (100-106, GeminiBio) and 1% penicillin/streptomycin at 37°C in a humidified 5% CO_2_ incubator. To polarize cells into M1 macrophages, GM-CSF (300-03, PeproTech), IFNγ (300-02, PeproTech), IL5 (200-06, PeproTech), and lipopolysaccharides (L4516-1MG, Sigma Aldrich) were used at 20 ng/mL. For M2 macrophage polarization, the cytokines used were M-CSF (300-25, PeproTech), IL4 (200-13, PeproTech), IL6, and IL13 (200-13, PeproTech) at 20 ng/mL. Cells were treated with various doses of DON (0.5–5 µmol/L) either from day 0 during the differentiation and polarization phase or from day 5 during the polarization phase until day 9.

### Phagocytosis assays

The *in vitro* phagocytosis assay was performed using fluorescence microscopy. Briefly, *in vitro* cultured macrophages were stained with PKH26 dye (PKH26GL-1KT, Sigma-Aldrich), and PC3 cells were labeled with 2.5 µmol/L carboxyfluorescein succinimidyl ester (CFSE; C34554, Thermo Fisher Scientific), according to the manufacturer’s instructions. Peripheral blood mononuclear cell (PBMC)–derived differentiated macrophages were treated with the vehicle or nontoxic dosage of DON either during differentiation days 1 to 9 or during polarization days 5 to 9 and were subsequently cocultured with PC3 prostate carcinoma cells at a 1:2 ratio (macrophages: PC3 cancer cells) and incubated for 2 hours at 37°C in sterile glass slides in six-well plates. The cells were washed repeatedly to remove nonphagocytosed PC3 cells and were subsequently imaged using an Echo Revolve microscope. We also used flow cytometry–based quantification of phagocytosis by PC3 cells. Briefly, unstained differentiated macrophages were cocultured with CFSE-labeled PC3 cells for 2 hours. Cells were repeatedly washed to remove nonphagocytosed cells, detached, and stained with viability dye, anti-CD45, and anti-CD11b for flow cytometry–based evaluation. To perform the *in vivo* phagocytosis assay, MB49-RFP or RM1-RFP cells were implanted for tumor development, following which the animals were treated with JHU083, as described earlier. The percentage of MB49-RFP^+^ or RM1-RFP^+^ tumor cells was quantified by flow cytometry–based evaluation in TAMs or subpopulations.

### Endothelial tube formation assay

Matrigel (354234, Corning) was thawed at 4°C overnight, then added to a 48-well plate and incubated at 37°C in 5% CO_2_ for 2 hours to solidify. Human monocyte-derived macrophages (HMDM; see “DON treatment in human PBMC-derived macrophages”) were harvested using cell stripper buffer (25-056-CI, Corning), counted, and then stained with Alexa Flour 488 antihuman CD206 at 1:200 (321114, BioLegend). HUVEC2 cells were stained using Alexa Fluor 594 antihuman CD31 at 1:200 (303126, BioLegend). Stained HUVEC2 cells were cocultured with macrophages at a 2:1 ratio for 16 hours on solidified Matrigel in human large vessel endothelial cell basal medium supplemented with a low serum growth supplement kit as described in “Tumor models and cell lines.” The culture plate was imaged using an Echo Revolve microscope. The acquired images were analyzed by ImageJ.

### IVIS imaging and quantification

Bioluminescence imaging of anesthetized mice was performed using the IVIS Lumina Series IV (PerkinElmer). Mice were intraperitoneally injected with d-luciferin (150 mg/kg). Images were acquired between 5 and 15 minutes after injection of D-luciferin, according to the standard operating procedure of the rodent imaging facility. The luciferase signal intensity total flux (p/s) was measured using Living Image software (PerkinElmer). MB49-luciferase RFP cells were used for these experiments (see “Tumor models and cell lines”).

### Immunohistochemistry

Immunostaining of MB49 and B6CaP tumor tissues was performed at the Oncology Tissue Services Core of Johns Hopkins University. Chromogenic immunolabeling was performed on formalin-fixed, paraffin-embedded sections using a Ventana Discovery Ultra autostainer (Roche Diagnostics). Briefly, after dewaxing and rehydration on board, epitope retrieval was performed using Ventana Ultra CC1 buffer (6414575001, Roche Diagnostics) at 96°C for 64 minutes or using a target retrieval solution (S170084-2, Dako) for 48 minutes at 96°C for CD8 antigen. Primary antibodies were used for staining and were detected using an antirabbit HQ detection system (7017936001 and 7017812001, Roche Diagnostics), followed by the ChromoMap DAB IHC detection kit (5266645001, Roche Diagnostics), counterstaining with hematoxylin, bluing, dehydration, and mounting. Whole slide brightfield scans were performed using a Hamamatsu NanoZoomer XR (C12000-02). Image analysis was performed using HALO ver. 3.3.2541 (Indica Labs) with module Area Quantification v2.1.10. The antibodies and dilutions used for IHC are listed in Supplementary Table S2.

### CD8^+^ T cell suppression assay

To perform cell suppression assays, 0.7 × 10^5^ fully differentiated and polarized M1 and M2 macrophages were harvested using cell stripper solution (25-056-CI, Corning) and plated into 24-well flat-bottom plates in 500 μL RPMI 1640 medium supplemented with 10% heat-inactivated FBS. CD8^+^ T cells were isolated and enriched from the PBMCs of the matched healthy donor using a human CD8^+^ T-cell–negative selection kit (19053, Stem Cell Technology). Negatively selected, enriched CD8^+^ T cells were stained with CellTrace Violet dye (C34557, Thermo Fisher Scientific) at a 1:1,000 dilution according to the manufacturer’s protocol. Then, 1.5 × 10^5^ CellTrace Violet-stained CD8^+^ T cells were resuspended in 500 μL RPMI 1640 medium supplemented with 10% heat-inactivated FBS and cocultured with previously seeded and adhered macrophages. Subsequently, 24 µL of Human CD3/CD28/CD2 T-Cell Activator (10970, Stem Cell Technology) and 30 unit/mL animal-free human recombinant IL2 (AF-200-02, PeproTech) were added, followed by incubation at 37°C and 5% CO_2_ for 3 days. CD8^+^ T cells were harvested at the endpoint, and cell proliferation and cytokine production were evaluated using flow cytometry.

### MTS assay

Either 10,000 or 20,000 MB49 cells were seeded in a 96-well flat-bottom culture plate per well and in DMEM supplemented with 10% FBS and 1% penicillin/streptomycin and incubated for 2 hours at 37°C and 5% CO_2_. Subsequently, the cells were either nontreated or treated with DON at 0.5, 1, or 2 µmol/L overnight. Then, 20 µL of the CellTiter 96 AQueous One Solution Cell Proliferation Assay (G3582, Promega) was added to each well and incubated at 37°C for 1 hour. Absorbance was measured at 490 nm using a SpectraMax Plus (Molecular Devices).

### Immunoblotting

Tumors were harvested at the experimental endpoint and digested to prepare single cells, as described above (see “Tumor digestion, flow cytometry, and fluorescence-activated cell sorting)”. Both CD45^+^ and CD45^−^ cells were isolated using a mouse CD45 tumor-infiltrating lymphocytes (TIL) MicroBeads Kit (130-110-618, Miltenyi), according to the manufacturer’s protocol. For the CD45^−^ cell fraction, dead cells were removed using the Dead Cell Removal Kit (130-090-101, Miltenyi). Whole cell protein lysates were collected using 1× MAPK buffer supplemented with protease inhibitors (SelleckChem and Sigma), and the protein concentration was determined using a Bradford reagent (5000202, Bio-Rad). Equal amounts of protein samples were loaded and resolved by SDS-PAGE and subsequently transferred to polyvinylidene difluoride membranes, followed by blocking with 5% BSA in Tris-buffered saline containing 0.1% Tween 20 (TBST). Membranes were incubated with primary antibodies (see Supplementary Table S3) overnight. Either StarBright 520 conjugated secondary antibody (12005870, Bio-Rad) or the HRP-conjugated secondary antibody (7074S, Cell Signaling Technology) were used to incubate the membrane. For HRP-conjugated secondary antibody incubated blots, enhanced chemiluminescence (Amersham) substrate (6883s, Cell Signaling Technology), as per the manufacturer’s protocol, was added and imaged using the ChemiDoc Imaging System (Bio-Rad).

### Statistics

Generating graphs and statistical analyses was performed with Prism 9 (GraphPad). Data are presented as mean values ± SEM, unless stated otherwise. Statistical analyses between two means were performed using an unpaired *t* test or a nonparametric two-tailed Mann–Whitney *t* test. Comparisons between three or more means were performed using a two-way ANOVA with a Bonferroni posttest. scRNA-seq and bulk RNA-seq DEG analysis were performed as described in the individual methods and figure legends. We performed log-rank (Mantel–Cox) tests for survival data (^∗^, *P* < 0.05; ^∗∗^, *P* < 0.01; ^∗∗∗^, *P* < 0.001; ^∗∗∗∗^, *P* < 0.0001).

### Data availability

The Kfoury and colleagues (41) prostate cancer bone metastasis dataset, along with its annotations, is publicly available through the GEO (accession number GSE143791). Bulk and single-cell RNA-sequencing data of B6CaP tumor data generated from this study have been deposited through the GEO (accession number GSE230162). All other data generated in this study are available within the article, and its Supplementary Data files or upon request from the corresponding author.

## Results

### Bone metastatic prostate cancer tumors contain TAMs enriched in glutamine-utilizing enzymes

**Figure 1.**
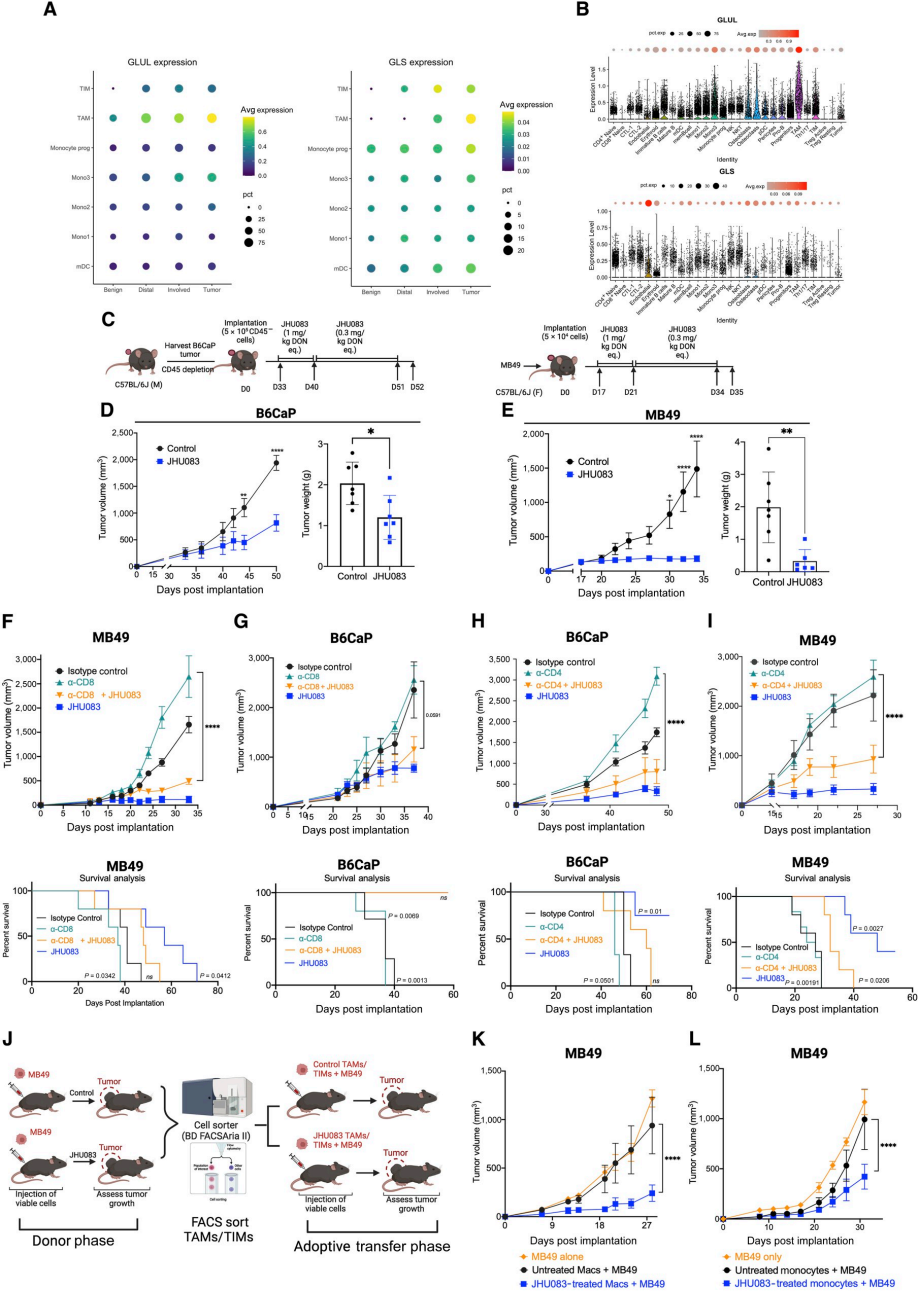
The antitumor activity of the glutamine antagonist JHU083 against urologic tumors is myeloid dependent. **A,** Dot plots showing expression levels and fractional abundance of myeloid cells expressing glutamine metabolism enzymes, *GLUL,* and *GLS* across samples (tumor, involved, distal, and benign). **B,** Violin plots showing the expression levels and fractional abundance of different cell types expressing *GLUL* and *GLS*. Data for **A** and **B** were generated using the previously published and publicly available scRNA-seq integrated dataset from nine metastatic prostate cancer and seven benign BM control patient samples by Kfoury and colleagues (41). **C,** Schematic diagrams showing the heterotopic syngeneic urologic tumor (B6CaP and MB49) models and therapeutic JHU083 treatment strategy. **D** and **E,** Tumor volume and tumor weight measurements (post-necropsy) in the B6CaP (*n* = 8/group) and MB49 (*n* = 9 or 10/group) tumors. **F** and **G,** Effects of CD8^+^ T cell depletion on tumor volume and animal survival for (**F**) MB49 (*n* = 4–5/group) and (**G**) B6CaP (*n* = 4–6/group) tumors, respectively. **H** and **I,** Effect of CD4^+^ T cell depletion on tumor volume of (**H**) MB49 (*n* = 4–5/group) and (**I**) B6CaP (*n* = 3–5/group) tumors and animal survival. Antitumor activity of JHU083-reprogrammed and adoptively transferred (**J)** TAMs (CD45^+^ Ly6G^−^ CD3^−^ CD11b^+^ F4.80^+^; *n* = 5 or 10/group) and (**K**) TIMs (CD45^+^ Ly6G^−^ CD3^−^ Ly6C ^(hi)^; *n* = 4 or 7/group) on MB49 tumor volume (**L**). Briefly, live, FACS-sorted TAMs or TIMs were mixed in a 1:1 ratio with cultured MB49 cells and were subsequently implanted on flanks to generate tumors in recipient mice. Data are represented as mean values ± SEM. All experiments were independently validated, at least in two separate biological replicates. Statistical analyses were performed using either *t* test or a two-way ANOVA using Bonferroni’s multiple comparisons (*, *P* < 0.05; **, *P* < 0.01; ***, *P* < 0.001; ****, *P* < 0.0001). Log-rank (Mantel–Cox) tests were performed for survival analysis (**F,** JHU083 relative to control, *P* = 0.0412; α-CD8b relative to α-CD8b + JHU083, *P* = 0.0342; α-CD8b + JHU083 vs. JHU083, *P* = ns. **I,** JHU083 vs. control, *P* = 0.01; α-CD4 vs. α-CD4 + JHU083, *P* = 0.0801; α-CD4 + JHU083 vs. JHU083, *P* = ns).

First, we used a published scRNA-seq data set from Kfoury and colleagues (41) to examine expression levels of key enzymes involved in glutamine utilization, synthesis, and transport, as well as enzymes involved in glycolysis, in human bone marrow mCRPC tumors and control tissues. In the bone metastatic tumor fraction, we observed an increased average expression of glutamine transporter *SLC1A5* (*ASCT2*), glutamine utilizing enzymes *GLS*, glutamine-fructose-6-phosphate transaminase (*GFPT1*), phosphoribosyl pyrophosphate amidotransferase (*PPAT*), glutamine synthetase (*GLUL*), and glycolytic enzyme hexokinase (*HK2*) relative to the benign or distal bone marrow (BM) fraction. (Supplementary Fig. S1A). Specifically, we observed increased expression of *GLUL* and *GLS* in TAMs (*CD68*^*+*^ and *CD163*^+^), TIMs (*LER*^*+*^ and *PLAUR*^*+*^), Mono 3 (monocyte cluster 3; *MNDA*^*+*^ and *CTSA*^+^), osteoclasts (*MMP9*^*+*^ and *SPP1*^*+*^), and endothelial cells (*GNG11*^*+*^ and *IFI27*^*+*^) in the tumor or involved BM fractions, suggesting elevated glutamine metabolism in these cell types (Fig. 1A; Supplementary Fig. S1B). Increased *GLUL* expression was found in TAM and Mono 3 clusters in the tumor BM relative to the distal, benign, and involved fractions (Fig. 1A and B). In addition, *GLS* expression was higher in the TAM cluster within the tumor fraction, suggesting a predominant glutamine metabolism activity in these tumor-infiltrating myeloid cells (Fig. 1A). We also found increased *GLS* expression in the Mono 3 cluster of the involved BM relative to the distal, benign, or tumor fractions, as well as increased *GLS* expression in other cell types such as endothelial cells and osteoclasts (Fig. 1A and B). Together, our findings indicate that key glutamine-metabolizing enzymes are abundant in the immunosuppressive and prometastatic TAM populations in metastatic prostate cancer tumors. The results also highlight a rationale for the multifaceted therapeutic benefit of utilizing a glutamine mimic to inhibit diverse glutamine-utilizing enzymes to metabolically alter these cells and thus increase the antitumor response (22).

### Glutamine antagonism with JHU083 shows potent antitumor activity in urologic tumors

Next, we employed two established syngeneic, heterotopic, and immunocompetent mouse models to investigate whether JHU083-induced glutamine antagonism can enhance host antitumor immunity in urologic tumors. As shown in Fig. 1C, preclinical subcutaneous B6CaP (prostate carcinoma; ref. 42) and MB49 (carcinogen-induced urothelial carcinoma; ref. 43) tumor models were treated with either vehicle or JHU083. Following the growth of palpable tumors (100–500 mm^3^), tumor-bearing animals were randomized into two cohorts. They were given either placebo or oral JHU083 (∼1 mg/kg DON equivalent) for 5 to 9 days, followed by a lower dose of (0.3 mg/kg DON equivalent; ref. 26). Significant TGI and tumor weight reduction were observed in both tumor types following JHU083 monotherapy (Fig. 1D and E) without any detectable consequential body weight loss (a measure of toxicity; Supplementary Fig. S1C). We further validated the JHU083-mediated robust TGI in another aggressive syngeneic murine prostate tumor carcinoma model, RM1 (44) (Supplementary Fig. S1D). Together, these results show that JHU083, administered as monotherapy, has potent antitumor activity, as evidenced by robust inhibition of the growth kinetics of urologic tumors without significant host toxicity.

### Antitumor activity of JHU083 in urologic tumors is only partially dependent on T cells

We next investigated the role of T cells in the observed antitumor efficacy of JHU083 in urologic tumors. To this end, we first depleted mice of CD8^+^ T cells using CD8β-specific depletion or isotype control antibodies (Supplementary Fig. S1E) and treated them with JHU083 or a vehicle. We observed aggressive tumor growth following antibody-mediated CD8^+^ T cell depletion in MB49 tumor-bearing animals (Fig. 1F), a phenotype that was less obvious in mice bearing B6CaP tumors (Fig. 1G). However, in both tumor models, JHU083 treatment caused a robust TGI despite CD8^+^ T cell depletion (Fig. 1F and G), suggesting that the antitumor activity of JHU083 is only partially dependent on CD8^+^ T cells in both tumor models.

Next, we investigated the role of CD4^+^ T cells in both urologic tumor models using CD4-specific depletion or isotype control antibodies. We observed that the depletion of CD4^+^ T cells resulted in increased tumor growth in the B6CaP tumor model (Fig. 1H). At the same time, there was no evident change in MB49 tumor growth (Fig. 1I), suggesting that CD4^+^ T cells do not restrict MB49 tumor growth. Like the responses observed following CD8^+^ T cell depletion, JHU083 treatment maintained antitumor efficacy even after CD4^+^ T cell depletion. Additionally, even though JHU083-treated animals survived longer than isotype control-treated animals, we observed no significant changes between JHU083 treatment alone and JHU083 treatment in CD4^+^ T cell–depleted tumors (Fig. 1H and I). Altogether, these results suggest that the efficacy of JHU083 in these urological tumor models is only partially dependent on CD4^+^ T cells and CD8^+^ T cells.

### JHU083-treated TAMs/TIMs mediate TGI in urologic tumors

We then investigated the role of myeloid cells in mediating the antitumor efficacy of JHU083 in urologic tumors. Since cancer cells are known to be highly dependent on glutamine metabolism (22), we hypothesized that JHU083 likely inhibits tumor growth through a broad mechanism of action, including both direct antitumor effects and indirect immune reprogramming effects. To separate these intertwined mechanisms of action for JHU083, we tested and tried to deplete macrophages. First, we identified and tried to optimize the - depletion of macrophages in the spleen, as previously shown (45). Although anti-CSF1R was able to deplete all CSF1R^+^ cells in the spleen, we observed a discrepancy in the effectiveness of macrophage ablation in the spleen, with only approximately 60% ablation achieved (Supplementary Fig. S1F). Orthogonally, we depleted macrophages using liposome encapsulated clodronate, a well-established technique for depleting tissue macrophages (46). As expected, we found a delayed response to JHU083 in MB49-bearing mice that continuously received clodronate during the experiment (Supplementary Fig. S1G).

To understand the effect of TAMs on tumor cells more conclusively, we used an orthogonal adoptive transfer (AT) design that exclusively models JHU083 reprogramming of myeloid cells (Supplementary Fig. S1H). Briefly, “donors” were implanted with MB49 and treated with either JHU083 or vehicle, then these tumor specimens were resected and viable TAMs (CD45^+^CD3^−^Ly6G^−^CD11b^+^F4/80^+^) were sorted, combined with fresh *in vitro* cultured MB49 cells at a 1:1 ratio, and implanted in the flanks of syngeneic “recipients” (Fig. 1J and K). A significant delay in MB49 tumor progression was observed in recipient animals that received JHU083-treated TAMs compared with recipient animals that received vehicle-treated TAMs (Fig. 1K), indicating that JHU083-reprogrammed TAMs mediate TGI. No significant change in tumor volume was observed when MB49 control tumors were compared with vehicle-treated AT macrophages. Altogether, these results support the antitumor role of JHU083-treated TAMs. This, in turn, could be due to phenotypic changes induced by JHU083-mediated reprogramming, which confers a potent antitumor state on TAMs within the TME. Using the same experimental design (Fig. 1J), we investigated the contribution of TIMs (live CD45^+^CD11b^+^CD3^−^Ly6G^−^Ly6C^high^) to TGI following JHU083 treatment (Supplementary Fig. S1H). A delayed TGI was observed in recipients receiving JHU083-treated TIMs than in recipients receiving vehicle-treated TIMs (Fig. 1L). Additionally, to understand the differences between JHU083 drug pressure post-AT and the reprogrammed TAMs (after JHU083 treatment in the donor experiment), we had two additional arms (*n* = 5) in which either vehicle-treated TAMs or JHU083-treated TAMs mixed with tumor cells received drug (JHU083) in the recipient animals. We observed no difference between drug-pressure-induced TGI versus no drug pressure, but just the JHU083 reprogrammed TAMs-induced TGI (Supplementary Fig. S1I). Altogether, these results demonstrate that JHU083 reprograms TAMs and TIMs, which then mediate antitumor effects in urological cancers.

### JHU083 reprograms immunosuppressive TAMs and TIMs in the TME to make them more inflammatory

**Figure 2.**
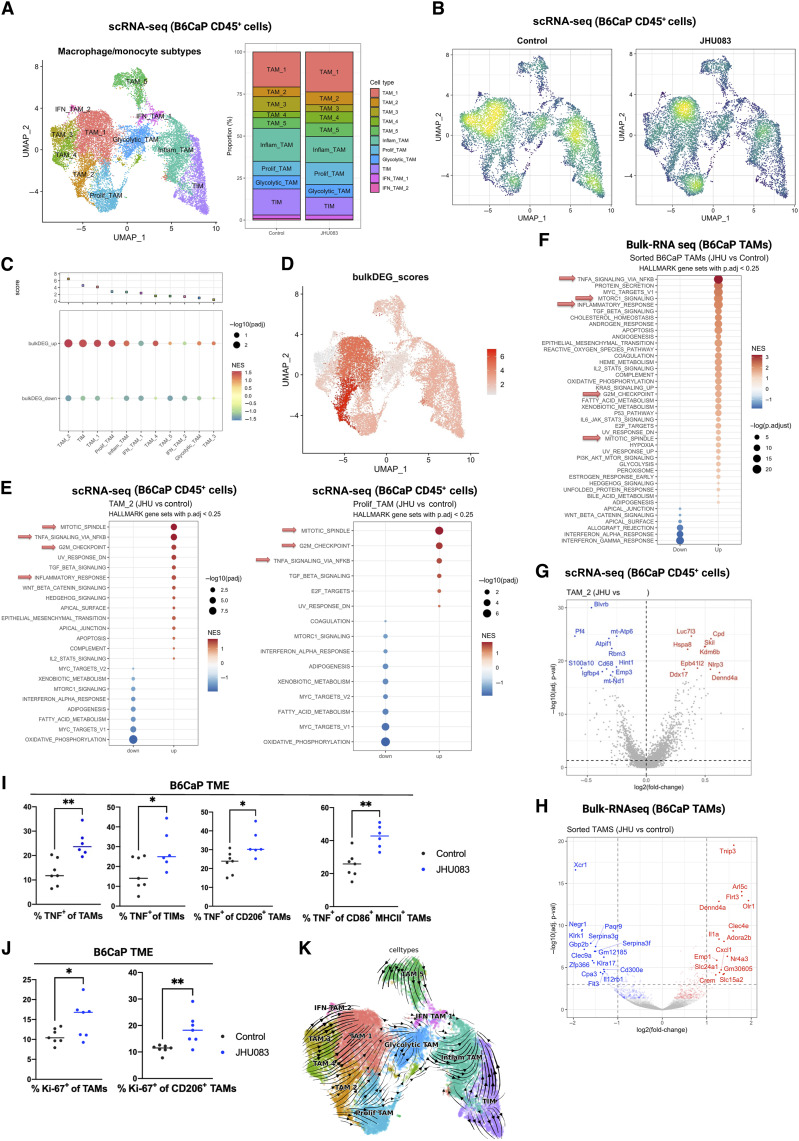
JHU083-mediated glutamine antagonism reprograms TME, differentially induces TAMs, TIMs, inflammatory TNF signaling and proliferation in TAMs/TIMs. **A,** UMAP and stacked bar plots showing the diversity and differential abundance of TAMs and monocyte (TIM) clusters in B6CaP tumors at an early point (day 7 posttreatment) using scRNA-seq (*n* = 6) across samples. UMAP, uniform manifold approximation and projection for dimension reduction. **B,** Density plots of different TAM and TIM clusters in control and JHU083-treated B6CaP tumors (identified in **A**). **C,** DEG scores were calculated for each TAM cluster based on the GSEA of significant DEGs from bulk RNA-seq of JHU083-treated vs. control TAMs (day 14 posttreatment, late time point; *n* = 6/group). **D,** UMAP plot based on the DEG scores identified using bulk RNA-seq of B6CaP-TAMs from **C**. UMAP, uniform manifold approximation and projection for dimension reduction. **E,** GSEA between JHU083-treated vs. control cells from the proliferative TAMs and TAM2. **F,** GSEA between bulk RNA-sequenced JHU083-treated TAMs and control TAMs sorted from B6CaP tumors (day 18 posttreatment; *n* = 6/group). **G,** Volcano plot of top DEGs from the TAM2 cluster between JHU083-treated vs. control. **H,** Volcano plot of top DEGs between JHU083-treated vs. control samples from bulk RNA-seq data. **I,** Intracellular quantification of the percentage (%) TNF^+^ population in TAMs and TIMs in B6CaP tumors. **J,** Intracellular expression of percentage (%) Ki-67^+^ TAMs in JHU083-treated TAMs vs. control B6CaP tumors, and (**K**) clusters identified from **A** overlaid using RNA velocity analysis. The root cells are the undifferentiated cells, and the developmental endpoints are the differentiated cells connected via arrows pointing to the likely developmental paths. Briefly, DEGs were calculated with DESeq2 in the bulk RNA-seq data, and with the Wilcoxon rank-sum test for the scRNA-seq data. The data are presented as mean values ± SEM. Statistical analyses were performed using either *t* test or a two-way ANOVA using Bonferroni’s multiple comparisons (*, *P* < 0.05; **, *P* < 0.01; ***, *P* < 0.001; ****, *P* < 0.0001).

We investigated the transcriptional responses of TAMs and TIMs in the prostate cancer TME in response to JHU083 treatment. As tumor microenvironments are a dynamic tissue subject to remodeling after immunotherapeutic intervention, we wanted to compare the transcript kinetics in TAMs from day 7 of JHU083 treatment (early time point) performed by scRNA-seq and contrast with the data from day 14 of JHU083 treatment (termed “late time point”) performed by bulk RNA-seq on FACS isolated macrophages (Supplementary Fig. S2A and S2B). After 7 days of JHU083 treatment (early time point), we identified the major immune compartments across both control and JHU083 treated animals (Supplementary Fig. S2C), indicating that JHU083 treatment does not induce inclusion or exclusion of a particular immune cell type. However, JHU083 treatment notably increased the intratumoral macrophage population (*Adgre1*^*+*^*Mrc1*^*+*^*Itgam*^*+*^; Supplementary Fig. 2SD). Given this shift toward macrophages, we then focused on the monocyte/macrophage populations and identified 10 unique TAM clusters and one TIM (*Ccr2*^*+*^*Ly6c2*^*+*^*Cd44*^*+*^) cluster (Fig. 2A; Supplementary Fig. 2E). Among the 10 clusters, we identify (i) inflammatory TAMs (Inflam_TAM; *S100a6*^*+*^*S100a4*^*+*^*S100a11*^*+*^); (ii) proliferating TAMs (Prolif_TAM; *Top2a*^*+*^*Pclaf*^*+*^*Diaph3*^*+*^); (iii) glycolytic TAMs (Glycolytic_TAM; *Slc2a1*^*+*^*Tpi1*^*+*^*Gpr137b*^*+*^), two type I IFN-responsive TAMs; (iv) IFN_TAM1 (*Ifit2*^*+*^*Isg15*^*+*^*Rsad2*^*+*^); and (v) IFN_TAM2 (*Iigp1*^*+*^*Gbp2*^*+*^*Ifi47*^*+*^), as well as five other TAM clusters named (vi) TAM1 (*Cd83*^*+*^*Sash1*^*+*^*Slc9a9*^*+*^), (vii) TAM2 (*Ccnb2*^*+*^*Birc5*^*+*^*Cenpa*^*+*^), (viii) TAM3 (*Ophn1*^*+*^*Itm2b*^*+*^*Fmd4b*^*+*^), (ix) TAM4 (*Nup210I*^*+*^*Pde4c*^*+*^*Pdpk1*^*+*^), and (x) TAM5 (*Tmsb4x*^*+*^*Cdk8*^*+*^*Rplp1*^*+*^). We specifically queried which of these populations accounted for the increase in the overall macrophage compartment and identified an increase in TAM1, TAM2, TAM4, TAM5, and proliferative TAMs and a decrease in TIMs, inflammatory TAMs, and TAM3 (Fig. 2A and B). We identified TAM2, TIM, TAM1, and Prolif_TAM as putative populations that expanded to dominate the transcriptional changes observed in the bulk RNA-seq data at day 14 (Fig. 2C and D). However, TIMs and the inflammatory TAM population, contrary to the early time point, also showed an expansion on day 14 post JHU083 treatment. This suggests a time-dependent influx of monocytes or expansion of inflammatory TAMs after JHU083 treatment (Fig. 2A–D) to explain the deviations observed.

Next, to understand how pathways are regulated in response to JHU083 treatment, we performed GSEA on each macrophage cluster from the early timepoint experiment. We identified four hallmark pathways enriched in the top scoring TAM clusters: (i) TNFA signaling via NF-kB, (ii) inflammatory response, (iii) mitotic spindle, and (iv) G2/M checkpoint (Fig. 2E; Supplementary Fig. S2F). We reviewed the late timepoint bulk RNA-seq dataset and found the same enrichment of these four pathways in JHU083-treated TAMs in addition to hallmark mTORC1 signaling (Fig. 2F; Supplementary Fig. S2G). Specific evaluation of the top DEGs from macrophages in both experiments revealed significant upregulation of key inflammatory genes (*Il1a* and *Il1b*), myeloid chemoattractant (*Cxcl1*)*,* inflammatory lectin type innate-sensing receptors (*Clec4e* and *Olr1*), and inflammasome (*Nlrp3*) across different TAM clusters (Fig. 2G and H; Supplementary Fig. S2H). Taken together, we identified TAM subpopulations that showed upregulation of hallmark TNF signaling and inflammatory pathways and an increased proportion of proliferative TAMs following JHU083 treatment.

To validate whether inflammatory reprogramming of TAMs increased in both tumor models, we investigated the differential expression of canonical markers of myeloid reprogramming and inflammation by flow cytometry at a late time point (Supplementary Fig. S2I). After JHU083 treatment, we found that JHU083 caused a percentage decrease in F4/80^+^ TAMs (as opposed to the scRNA-seq data early point), while the percentage of CD11b^+^ cells and TIMs increased at a late time point (day 14) in B6CaP tumors (Supplementary Fig. S2J and S2K). Evaluation of F4/80^+^ and CD11b^+^ tissue areas in B6CaP tumors using IHC showed a similar change (Supplementary Fig. S2M). Concurrently, an increase in the percentage of both F4/80^+^ TAMs and Ly6C^(hi)^ TIMs was observed in MB49 tumors following JHU083 therapy (Supplementary Fig. S2N). Since there was a discrepancy between B6CaP and MB49 in terms of percentage TAM levels after JHU083 treatment, we investigated whether JHU083-induced changes were related to the intratumoral abundance of M1 (live CD45^+^Ly6C^−^Ly6G^−^F4/80^+^MHCII^+^CD86^+^) or M2 (live CD45^+^Ly6C^−^Ly6G^−^F4/80^+^CD206^+^) TAMs in both tumor types. After JHU083 treatment, we found an increase in M2 surface markers (CD206^+^) and no change in canonical M1 surface markers (CD86^+^ MHCII^+^) in B6CaP TAMs, in contrast to what was previously reported by Oh and colleagues in 4T1 murine tumors (26), highlighting that there are model-specific differences (Supplementary Fig. S2J–S2L).

Finally, as bulk RNA-seq experiments showed TNF signaling was enhanced after JHU083 treatment, we performed intracellular staining for TNF in TAMs and TIMs from B6CaP and MB49 tumors. The percentage of TNF^+^ cells and their expression increased in both M1 and M2 TAMs and TIMs after JHU083 treatment in B6CaP tumors (Fig. 2I; Supplementary Fig. S2L). In MB49 tumors, the percentage of TNF^+^ TIMs similarly increased (Supplementary Fig. S2N). Despite TNF^+^ TAM showing a decreasing trend, the intensity of intracellular TNF staining increased significantly in TAMs, especially in the M1 TAMs as shown by gMFI. This indicates a functional inflammatory reprogramming of TAMs, which is not reflected by an immediate reversal of canonical M2 (CD206) marker expression, highlighting the importance of functional assessment rather than surface phenotyping when determining the macrophage state intratumorally.

Overall, using multiple approaches to investigate different subclusters/populations of TAMs and TIMs and validation using both transcriptomics and flow cytometry, we established that JHU083 treatment increases TNF signaling and overall inflammatory signaling, findings that are consistent with the previously reported findings in the 4T1 model of breast cancer (26). We, therefore, hypothesized that the TAM-mediated antitumor effect observed in Fig. 1J is likely driven by JHU083-induced functional reprogramming of TAMs and TIMs, thus rendering them strongly tumor-reactive for a prolonged duration.

### JHU083 conditions a fraction of tumor-resident TAMs to proliferate

We also observed the enrichment of hallmark proliferation pathways (mitotic spindle and/or G2/M checkpoint) in several macrophage populations (TAM2, TAM1, TAM4, TAM5, inflammatory TAMs, and glycolytic TAMs) after JHU083 treatment at both early and late timepoints (Fig. 2E and F; Supplementary Fig. S2F). The proliferative TAM cluster, one of the most expanded populations at both early and late time points, was also most highly enriched in cell-cycle genes corresponding to G2/M transition genes (Supplementary Fig. S2O). Flow cytometry analysis confirmed that JHU083 treatment increased the percentage of Ki-67^+^ TAMs in the B6CaP TME, with CD206-coexpressing TAMs being the most strongly enhanced (Fig. 2J). We then investigated the ontogeny of proliferative TAM clusters from TIMs by performing RNA velocity analysis to predict the transcriptional trajectories of each cell (36). We deduced that the proliferative TAMs were developmentally unrelated to the infiltrating monocyte-derived populations within the B6CaP TME (Fig. 2K). These findings agree with previous pioneering studies highlighting the loss of the proliferative capacity of infiltrating monocyte-derived macrophages (47, 48). These data indicate that JHU083 treatment can affect cell-cycle signaling in intratumoral macrophages and may cause tissue-resident macrophages to proliferate. Knowing that JHU083 reprogramming of TAMs contributes to a delay in tumor growth, the observed overwhelming impact on proliferation becomes a key phenotype of JHU083-reprogrammed TAMs.

### JHU083 promotes phagocytosis and decreases angiogenesis in TME

**Figure 3.**
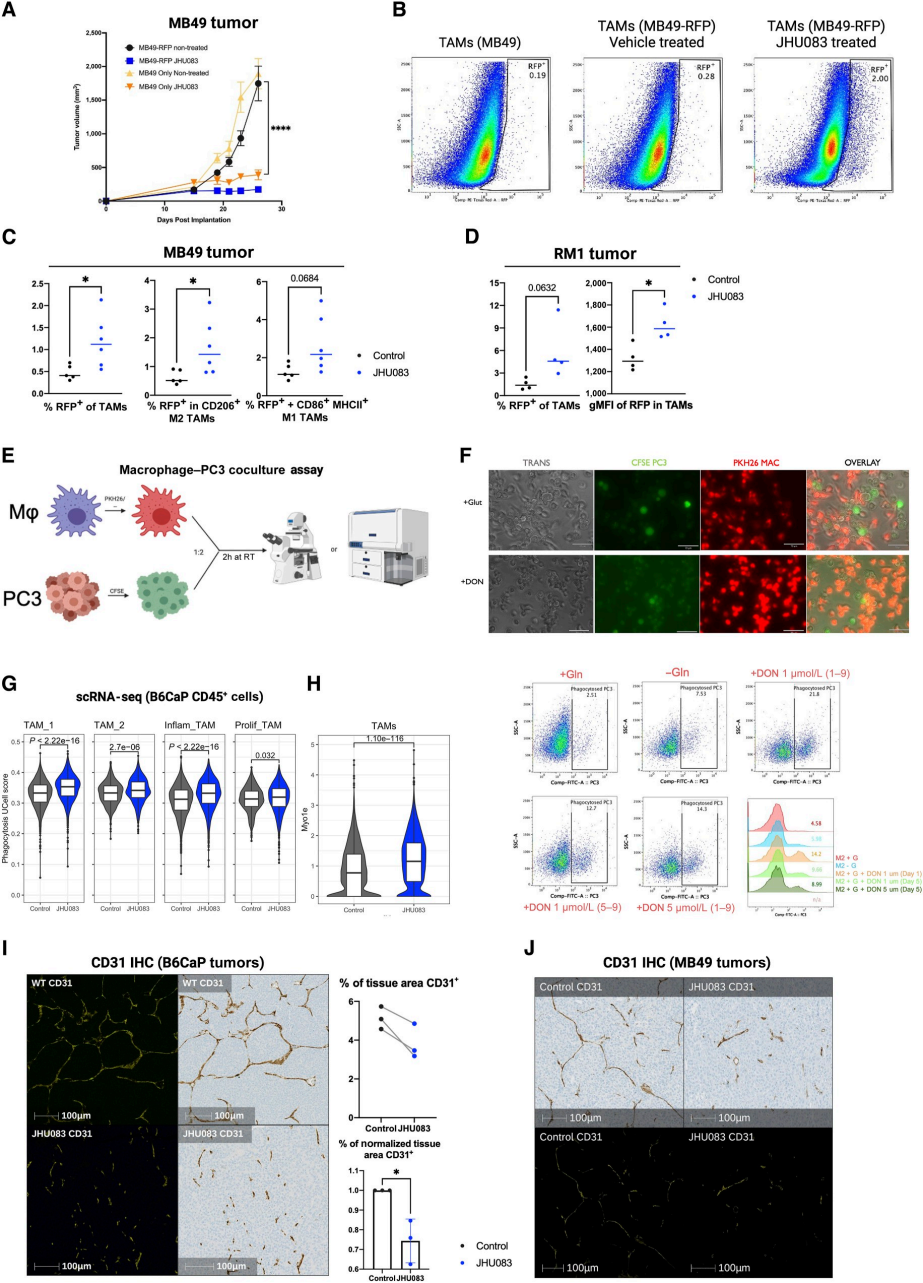
JHU083-induced glutamine antagonism promotes phagocytosis of tumor cells by TAMs and decreases angiogenesis in the TME. **A,** Tumor volume measurement of MB49 tumors in different treatment conditions. JHU083 treatment decreases tumor growth in MB49-RFP^+^ tumors relative to nontreated (*n* = 5 or 6/group) tumors. **B,** Representative flow cytometry plots of RFP^+^ MB49 tumor cells in TAMs in JHU083-treated vs. vehicle-treated tumors. **C,** Percentage of RFP^+^ cells within overall TAMs, CD206^+^ TAMs (M2-TAMs), and CD86^+^ MHCII^+^ TAMs (M1-TAMs) in MB49 tumors in control vs. JHU083-treated tumors. **D,** Percentage of RM1-RFP^+^ cells and corresponding mean fluorescence intensity (gMFI) of TAMs (*n* = 4/group) in control vs. JHU083-treated tumors. **E,** Schematic diagram showing the determination of the phagocytic activity of TAMs. Briefly, DON-treated, PBMC-derived macrophages were cocultured with CFSE-labeled PC3 cells, and phagocytic activity was determined using flow cytometry. **F,** Representative photomicrographs showing increased phagocytosis in DON-treated and PKH26-labeled macrophages (PKH26 MAC) cocultured with CFSE-labeled PC3 (CFSE PC3) by immunofluorescence microscopy and quantification using flow cytometry. **G,** Violin plots of phagocytosis UCell scores on TAM_1, TAM_2, Inflam_TAM, and Prolif_TAM (Wilcoxon rank-sum test) identified in scRNA-seq analysis of CD45^+^ cells from B6CaP tumors. **H,** Violin plot for *Myo1e* expression in all TAMs in B6CaP-derived TAMs identified in scRNA-seq analysis of CD45^+^ cells from B6CaP tumors. **I,** IHC-based quantification of CD31^+^ area intensity in B6CaP tumors (*n* = 3/group), and (**J**) IHC quantification of CD31 intensity in MB49 tumors. Statistical analyses were performed using *t* test or two-way ANOVA using Bonferroni’s multiple comparisons. Violin plots from scRNA-seq studies are analyzed using the Wilcoxon rank-sum test. (*, *P* < 0.05; **, *P* < 0.01; ***, *P* < 0.001; ****, *P* < 0.0001).

We next investigated the effects of JHU083 on phagocytosis (5) and angiogenesis (19), well characterized functional roles of TAMs. We first compared RFP^+^ and RFP^−^ MB49 cells to establish whether the expression of reporter proteins (RFP, luciferase) affected the immunogenicity of the cell line and determined using tumor volume measurements and *in vivo* imaging for luciferase activity that JHU083 treatment generated comparable TGI in both lines (Fig. 3A; Supplementary Fig. S3A). After JHU083 treatment, the *in vivo* TAMs were more phagocytic of tumor cells (Fig. 3B), and, importantly, this increased phagocytosis was observed in both CD206^+^ M2 TAMs and CD86^+^MHCII^+^ M1 TAMs (Fig. 3C; Supplementary Fig. S3B). We also observed increased tumor cell phagocytosis after JHU083 treatment in a prostate adenocarcinoma murine model (Fig. 3D; Supplementary Fig. S3C), confirming that the phenotypic response was consistent across different urologic tumors.

Next, we investigated whether JHU083 induced increased phagocytosis in intratumoral TAMs due to an increase in tumor cell phagocytosis or a direct effect on increasing the phagocytic capacity of the macrophages themselves. To tease this apart, we treated *in vitro* M2-like HMDMs (40) with concentrations of 1 or 5 µmol/L DON either during the differentiation phase (D1–D5) and/or during the polarization phase (D5–D9; Fig. 3E). While treatment with DON during the differentiation or polarization phase resulted in enhanced phagocytosis, treatment with DON during the entire process resulted in the highest increase in phagocytosis of PC3 cells compared to the untreated control, as measured by immunofluorescence and further quantified by flow cytometry (Fig. 3F). This result suggests that inhibition of glutamine metabolism in differentiated and polarized macrophages immediately augments their phagocytic activity. This phenotype was further supported when we examined phagocytosis gene scores within the scRNA-seq dataset in all TAM clusters. Specifically, TAM1, TAM2, Inflam_TAM, and Prolif_TAM showed an enriched UCell score for phagocytosis-related genes following JHU083 treatment (Fig. 3G). Moreover, *Myo1e*, a key late-stage phagocytic force generating the myosin-II gene (49), was highly expressed in TAMs of JHU083-treated B6CaP tumors (Fig. 3H).

These results show that targeting glutamine metabolism directly increases the phagocytic activity of TAMs against live tumor cells.

Additionally, DON inhibits glutamine synthetase activity (50), a proangiogenic enzyme known to promote metastasis (19). Therefore, we sought to determine the effect of JHU083 treatment on angiogenesis in the TME. We performed IHC for CD31, a vascular differentiation marker, in the B6CaP and MB49 tumors. After JHU083 treatment, we observed that the percentage of tumor tissue area stained for CD31 decreased significantly in tumors after excluding necrotic regions in B6CaP and MB49 tumors (Fig. 3I and J). To understand if the effect of decreased angiogenesis *in vivo* was due to macrophages, we performed an *in vitro* endothelial cell tube formation assay. We treated M2-like HMDMs with 2 µmol/L of DON during the early differentiation phase (days 1–5) or 5 µmol/L of DON during the polarization phase (days 5–9). Those macrophages were then harvested, washed, and cocultured with untreated HUVEC2 cells. Due to the low cell density, endothelial cells were only able to form incomplete tubes when cultured by themselves, but with M2 macrophages added, we observed well-formed capillary-like tubes, indicative of the proangiogenic function of M2 macrophages (Supplementary Fig. S3D). However, when M2 macrophages were exposed to DON during their differentiation and/or polarization, the cocultured endothelial cells became less capable of forming intact capillary-like structures compared to those cells cocultured with untreated macrophages, as shown by the quantification of total tube length, suggesting that the inhibition of glutamine utilization in M2 macrophages hampered their proangiogenic function (Supplementary Fig. S3E). These results suggest that glutamine blockade in TAMs via metabolic inhibition results in augmented phagocytosis and diminished tumor angiogenesis, two essential functional tumor control mechanisms.

### JHU083 induces parallel metabolic changes in TAMs

**Figure 4.**
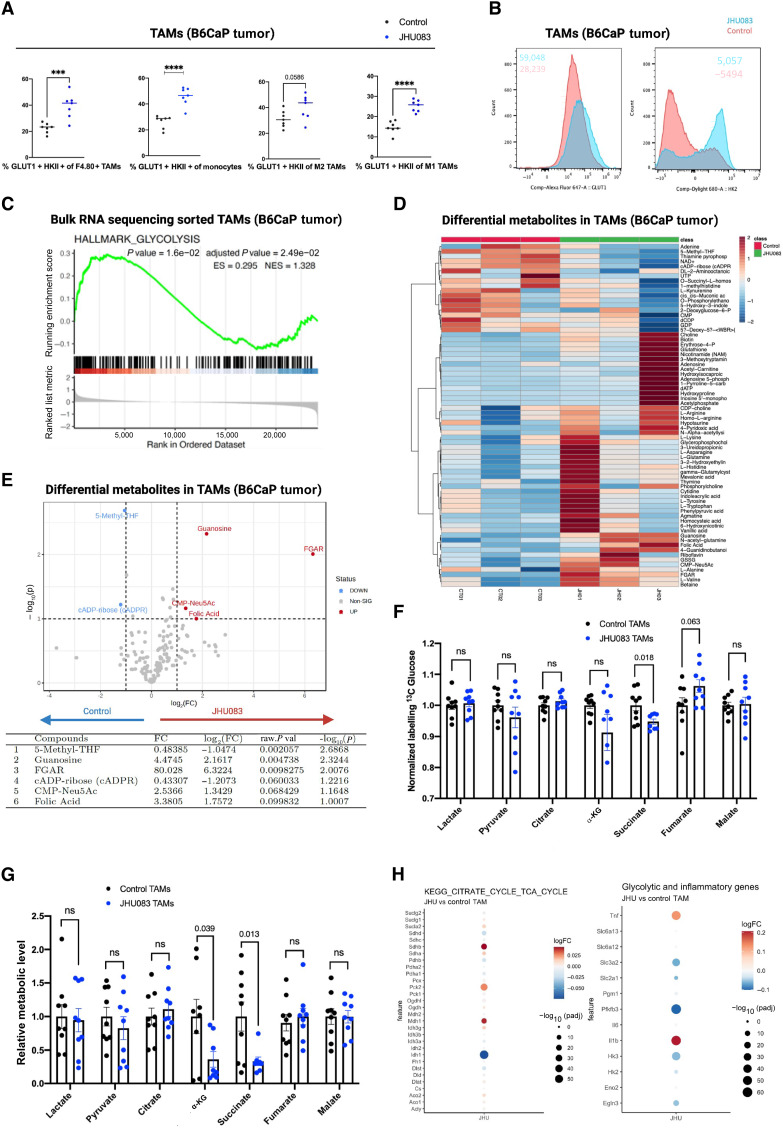
JHU083-induced glutamine antagonism caused a divergent metabolic response affecting glycolysis, purine metabolism, and succinate in TAMs. **A** and **B,** Surface and intracellular expression of GLUT1 and HKII (percentage positive population and mean fluorescence intensity) on B6CaP-derived TAMs. **C,** GSEA showing the enrichment of the Hallmark glycolysis gene set among DEGs in bulk RNA-sequenced FACS-sorted B6CaP-derived TAMs following JHU083 treatment relative to control. **D,** Heat map showing the differential metabolites in TAMs sorted from JHU083-treated and control B6CaP tumors (*n* = 3/group). **E,** Volcano plot showing log_2_ fold change vs. –log_10_ (FDR-corrected *P* value), representing fold changes in metabolite abundance in one-carbon metabolism, purine nucleotide metabolism, and hexosamine pathway in JHU083-treated vs. control TAMs. **F,** Normalized relative labeled metabolites from U-^13^C glucose in the TCA cycle in TAMs derived from B6CaP tumors (*n* = 9/group in two independent experiments). **G,** Normalized relative metabolite abundances in the TCA cycle in TAMs derived from B6CaP tumors (*n* = 9/group from two independent experiments) and (**H**) log fold-change of TCA cycle enzymes and inflammatory cytokine transcripts in TAMs (from scRNA-seq at an early time point (day 7 posttreatment). DEGs for GSEA in **C** were calculated with DESeq2, and statistics on genes of interest in **H** were calculated with the Wilcoxon rank-sum test. All other statistical analyses were performed using the unpaired *t* test (*, *P* < 0.05; **, *P* < 0.01; ***, *P* < 0.001; ****, *P* < 0.0001).

DON-mediated glutamine antagonism has broad-ranging effects on glutamine-consuming enzymes in multiple metabolic pathways and on glutaminolysis (22). Divergent metabolic reprogramming in T cells relative to cancer cells in the TME owing to differential effects of glutamine inhibition on the two cell types has been previously reported (27). Since we found that JHU083-mediated glutamine metabolism inhibition led to tumor suppression, concomitant with TAM inflammatory reprogramming, we hypothesized that blocking glutamine metabolism would significantly affect the metabolic milieu of both the TME and TAMs. We first investigated the expression of metabolic markers in B6CaP intratumoral TAMs and TIMs using flow cytometry. We did not observe significant changes in the relative abundance of TAMs expressing mitochondrial proteins voltage-dependent anion channel 1 (mitochondrial mass), TOMM20 (OXPHOS), or carnitine palmitoyl-transferase 1α (fatty acid oxidation) in JHU083-treated TAMs (Supplementary Fig. S4A). However, a marked increase in GLUT1 (glucose transporter) and HKII (glycolysis) was observed in TAMs and TIMs following JHU083 treatment (Fig. 4A and B). Elevated levels of GLUT1 and HK2 were most significant in CD86+MHCII+ M1 TAMs, whereas we still observed a trend toward elevated levels in CD206^+^ M2 TAMs (Fig. 4A). Indeed, GSEA of DEGs from the late timepoint RNA-seq dataset revealed significant enrichment of glycolytic pathway genes (Fig. 4C). Additionally, mTORC1, a key regulator of glycolysis (51), was found to be upregulated in JHU083-treated TAMs (Supplementary Fig. S2G).

Next, we investigated the direct effects of JHU083 on the metabolism of TAMs. The metabolic discrepancy between TAMs and homogenous *ex vivo*-generated macrophage populations was likely due to the highly complex milieu of theTME (2, 52). This prompted us to modify and optimize the rapid digestion, sorting, and tumor sample processing protocol (53) for TAMs to understand the metabolomic changes in these cells in the intratumoral milieu after JHU083 treatment (Supplementary Fig. S4B).

Using an LC-MS/MS–based targeted metabolomic approach, we quantified the relative abundance of 156 key metabolites in sorted TAMs from B6CaP tumors. Normalized differential metabolites from both JHU083-treated TAMs and control TAMs (*n* = 3/group) were used for metabolic quantification (Fig. 4D; Supplementary Fig. S4C). We discovered that purine nucleotide metabolism stalled with increased FGAR and guanosine in JHU083-treated TAMs (Fig. 4E). The purine synthesis pathway has recently been implicated in promoting TAM polarization to an immunosuppressive protumoral phenotype (54), which might explain its probable role in contributing towards inhibition-driven repolarization of JHU083-treated TAMs. Due to the technical challenges in recovering and missing the LC-MS peaks for lost metabolites in a targeted metabolomics approach, we had limited success quantifying glycolysis or tricarboxylic acid (TCA) cycle metabolites. This challenge was overcome by further optimizing our protocol and performing in vivo tracing of [U-^13^C] glucose (27) in rapid-sorted TAMs from B6CaP tumors to understand the effects of JHU083 on glycolysis and on the TCA cycle. We observed a decreased contribution of glucose carbons to succinate and an elevated level of glucose carbons in fumarate in JHU083-treated TAMs (Fig. 4F; Supplementary Fig. S4D). This could be due to the blockade of glutaminolysis, which is a major carbon source for succinate. To further understand the implications of a disrupted flux of glucose carbons between succinate and fumarate, we examined the relative abundance of all TCA cycle metabolites. We found a decreased abundance of both succinate and α-ketoglutarate (α-KG) in TAMs upon JHU083-mediated glutamine blockade, suggesting that blocking glutamine anaplerosis affects the overall levels of both metabolite responses, likely affecting TCA cycle intermediates (Fig. 4G). Our results indicated that a disrupted TCA cycle probably drives the proinflammatory phenotype, as previously described in homogenous *ex vivo* inflammatory M1 macrophages. M1 macrophages with low α-ketoglutarate levels, due to a disrupted TCA cycle, mediate blockade of HIF-1α hydroxylation and degradation, resulting in the induction of IL1β signaling (55, 56). To understand this regulation, we investigated TCA cycle enzymes and inflammatory cytokine transcripts in all TAMs using the scRNA-seq dataset (Fig. 4H). We observed increased succinate dehydrogenase subunit b (*Sdhb*) and *II1b* transcripts in B6CaP-TAMs following JHU083 treatment (Fig. 4H), as we expected. Moreover, bulk RNA-seq of sorted B6CaP-derived TAMs showed a highly enriched score for signaling by the interleukin pathway following JHU083 treatment (Supplementary Fig. S4E), suggesting increased proinflammation as a direct consequence of JHU083-induced divergent metabolic reshuffling in TAMs. In conclusion, these results indicate that JHU083 contributes to the intratumoral metabolic plasticity in prostate carcinoma TAMs and induces glycolysis to fuel a broken/disrupted TCA cycle, processes that may be partly responsible for inducing proinflammatory signaling. In parallel, glutamine antagonism affects purine nucleotide metabolism in TAMs in the TME, which may play a role in the polarization shift of TAMs.

### JHU083 affects tumor cell metabolism and induces cell death in urologic tumors

**Figure 5.**
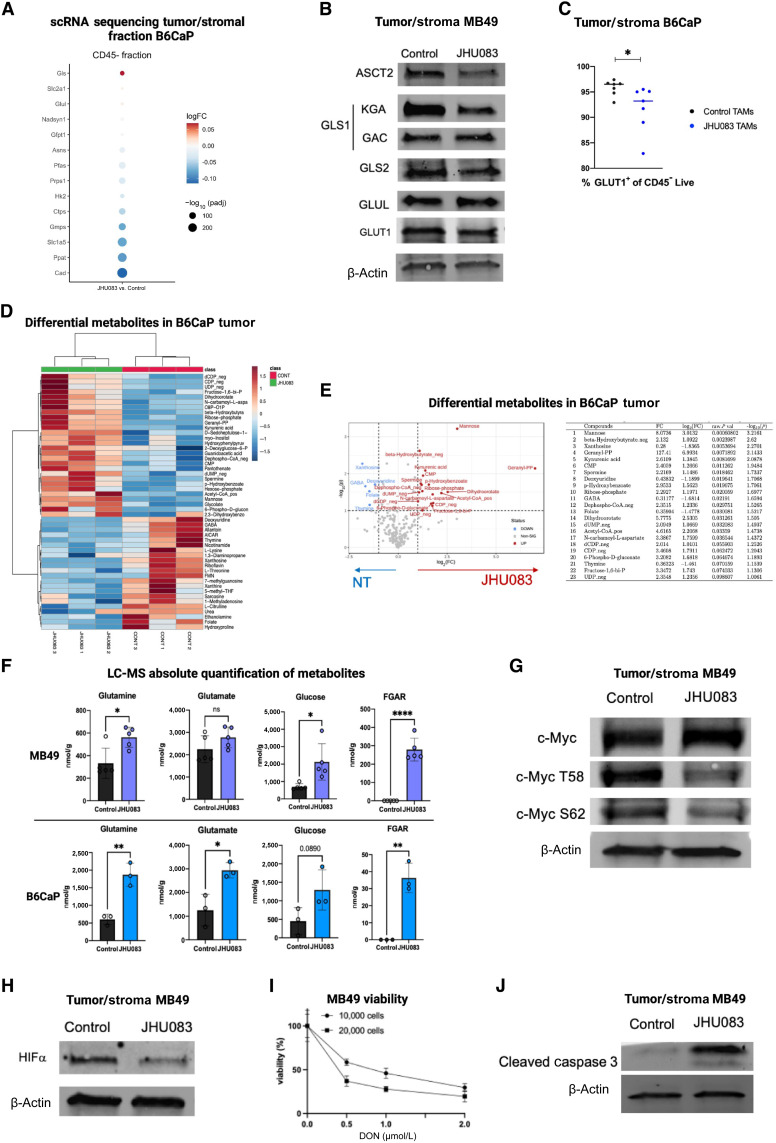
JHU083-induced glutamine antagonism affects tumor cell metabolism and induces cell death in urologic tumors. **A,** Log-fold changes of glutamine utilizing enzymes after JHU083 treatment vs. control tumors in CD45^−^ sorted cells from B6CaP tumors, followed by scRNA-seq. **B,** Western blot showing qualitative changes in the levels of glutamine synthesizing/utilizing enzymes and transporters in the CD45^−^ fraction of MB49 tumors. **C,** Percentage of GLUT1^+^ CD45^−^ live cells determined by flow cytometry in B6CaP tumors (*n* = 7/group). **D,** Targeted metabolomic analysis of B6CaP tumors by LC-MS/MS (*n* = 3/group). **E,** Volcano plot showing key metabolite levels of JHU083-treated vs. nontreated control tumors based on the metabolomic analysis shown in **D**. **F,** Absolute quantification of metabolites by LC/MS-MS (*n* = 3 or 5/group). **G** and **H,** Western blot images showing qualitative changes in c-MYC, phospho-c-MYC, and HIF-1ɑ in MB49 tumors following JHU083 treatment, and (**H** and **I**) MTT assay in DON-treated MB49 cells and immunoblot of cleaved caspase 3 quantification in CD45^−^ fraction MB49 tumors (**J**). Statistical analyses were performed using the unpaired *t* test. (*, *P* < 0.05; **, *P* < 0.01; ***, *P* < 0.001; ****, *P* < 0.0001).

Glutamine is a key carbon and nitrogen source for energy production and for nucleotide and amino acid synthesis (57). Previously, Leone and colleagues (27) showed that JHU083-mediated glutamine blockade results in suppressed oxidative and glycolytic metabolism in cancer cells, resulting in decreased hypoxia and ultimately nutrient depletion. However, these mechanisms have not been entirely elucidated in myeloid-rich urologic tumors (27). We were able to identify decreased expression of enzymes associated with glutamine transport (*Slc1a5*), nucleotide synthesis (*Cad*, *Ppat*, and *Gmps*), and a possible compensatory increase in *Gls* transcript levels after glutaminolysis blockade (Fig. 5A).

Next, we performed Western blot analyses of tumor/stromal cell lysates from MB49 and B6CaP tumors for key glutamine-utilizing enzymes following JHU083 treatment. JHU083 treatment suppressed levels of ASCT2 (a major glutamine transporter), glutaminase 1 (GLS1), KGA isoform, and glutaminase 2 (GLS2; Fig. 5B). While we did not observe a significant difference in GLUT1 levels in the whole cell lysate from MB49 tumor cells, we report reduced surface GLUT1 expression in the B6CaP tumor cells using flow cytometry (Fig. 5B and C).

Knowing that blocking glutamine in urologic tumor cells influences diverse pathways, we used a targeted, unbiased metabolomic screen using whole B6CaP tumors (Supplementary Fig. S5A). We quantified 218 tumor metabolites, of which 23 showed differential abundance (*P* < 0.01) between JHU083-treated and control B6CaP tumors (Fig. 5D), which were then subjected to metabolic pathway analysis (Supplementary Fig. S5B). Metabolic pathway analysis revealed severely reduced nucleotide metabolism metabolites (xanthosine, CMP, deoxyuridine, dUMP, dCDP, CDP, thymine, and UDP; Fig. 5E). Additionally, JHU083 treatment changed metabolite levels, resulting in impaired amino acid metabolism, one-carbon metabolism, glycolysis, the hexosamine pathway, and TCA cycle metabolism (Supplementary Fig. S5B). To better understand the net result of impaired metabolism in tumor/stromal cells, we used LC-MS–based absolute quantification of intratumoral glucose, glutamine, glutamate, and FGAR in both MB49 and B6CaP tumors. We observed an increase in glutamine, glucose, and FGAR (Fig. 5F), and inferred that these metabolites in the tumors could not be consumed.

As JHU083 is known to affect c-MYC and HIF-1α signaling (27, 57), we investigated c-MYC and HIF-1α expression levels in MB49 tumor/stromal cells (Fig. 5G and H). While we did not observe any change in total c-MYC levels after JHU083 treatment, we observed a significant decrease in phosphorylated c-MYC (both T58 and S62) and HIF-1α levels. These results suggested a global metabolic shutdown in urological tumor cells, prompting us to investigate the possible effects of JHU083 on tumor cell viability. We found a dose-dependent reduction in MB49 cell viability *in vitro* (Fig. 5I), confirmed by the enhanced cleaved-caspase-3 levels seen in tumor/stromal cells from JHU083-treated MB49 tumors (Fig. 5J). Unlike normal cells, which maintain a balance between catabolism and anabolism, rapidly proliferating tumor cells are chiefly anabolic (from glutaminolysis, glycolysis, and *de novo* fatty acid synthesis) to meet the ever-increasing bioenergetic needs and essential building blocks for rapid proliferation (58). Together, our results suggest a profound antitumor effect of JHU083 via impaired tumor cell metabolism, leading to disruption of HIF-1α and c-MYC signaling. This, in turn, likely causes a global metabolic shutdown, possibly driving the observed apoptosis in the tumors/stromal cells.

### JHU083 induces markers of long-lived T cells and affects immunosuppressive regulatory T cells in the TME

**Figure 6.**
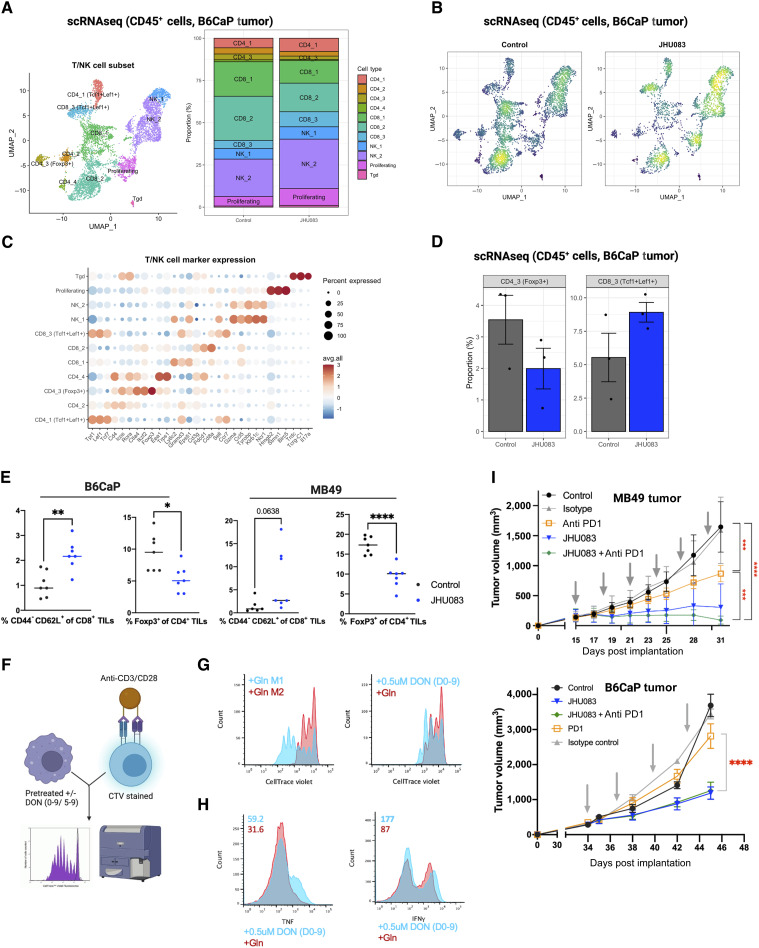
JHU083-induced glutamine antagonism causes extensive reprogramming of TILs. **A,** UMAP plot showing lymphocytic cell subsets identified from scRNA-seq analysis of CD45^+^ cells isolated from B6CaP tumors (JHU083-treated vs. control). **B,** UMAP density plots showing all NK and T cells. **C,** Dot plots of normalized expression of selected marker genes in T and NK cell subsets identified in **A**. **D,** Changes in proportions of NK cell subsets, stem cell–like CD8^+^ T cells, and CD4^−^ Tregs from scRNA-seq (*n* = 3/group). **E,** Surface and intracellular expression of stem-like CD8^+^ T cells and Foxp3^+^ percentage population in B6CaP and MB49 tumors (*n* = 7/8 per group). **F,** Schematic workflow of T cell coculture with DON-pretreated human primary macrophages. **G,** CD8^+^ T cell proliferation measurement using autologous CD8^+^ T cells isolated from human PBMCs and cocultured with monocyte-derived macrophages pretreated with DON either during differentiation (days 0–9) or during polarization (days 5–9) phase. **H,** gMFI of TNF and IFNγ in cocultured autologous CD8^+^ T cells with DON pretreated monocyte-derived macrophages, and (**I**) Tumor volume measurement of MB49 and B6CaP tumors during the therapeutic window. Briefly, following the development of palpable tumors, tumor-bearing C57BL/6J mice were injected every third day with anti-PD1 alone or in combination with daily oral gavage of JHU083. The data are presented as mean values ± SEM. Statistical analyses were performed using either *t* test or a two-way ANOVA using Bonferroni’s multiple comparisons (*, *P* < 0.05; **, *P* < 0.01; ***, *P* < 0.001; ****, *P* < 0.0001). UMAP, uniform manifold approximation and projection for dimension reduction.

JHU083 promotes antitumor immunity by conditioning TILs toward a long-lived, memory-like phenotype that is highly proliferative, markedly activated, and capable of enhanced effector function in the colon cancer TME (27). Despite observing only partial CD8^+^ and CD4^+^ T cell dependence in JHU083-mediated antitumor immunity in urologic tumor models (Fig. 1F–I), we hypothesized that JHU083 treatment would still cause functional changes in TILs from urological cancers, as reported previously in other models (27). We examined the early timepoint scRNA-seq dataset from B6CaP tumors (Supplementary Fig. S2A and S2B). We identified the presence of T cells (*Cd3d*^*+*^ and *Trbc2*^*+*^), NK cells (*Klrb1c*^*+*^, *Gzma*^*+*^, and *Ncr1*^*+*^), and ɣδ T cells (*Trdc*^*+*^ and *Il17a*^*+*^) in all samples, independent of JHU083 treatment (Supplementary Fig. S2C and S2D). Specific analysis of just the lymphoid cells identified 11 different clusters, including (i) CD4_1 (*Tcf*^+^ and *Lef1*^+^), (ii) CD4_2 (*Cd4*^+^ and *Icos*^+^), (iii) CD4_3 (*FoxP3*^+^, *Ikzf2*^+^, and *Ctla4*^+^), (iv) CD4_4 (*Cd4*^+^, *Eea1*^+^, and *Trps1*^+^), (v) CD8_1 (*Cd8a*^+^ and *Epsti1*^+^), (vi) CD8_2 (*Cd8a*^+^ and *Pdcd1*^+^), (vii) CD8_3 (*Cd8a*^+^, *Tcf1*^+^, and *Lef1*^+^), (viii) NK_1 (*Gzma*^+^, *Klrb1c*^+^, and *Ncr1*^+^), (ix) NK_2 (*Gzma*^+^, *Tyrobp*^+^, and *Ncr1*^+^), (x) proliferating (*Hmgb2*^+^, *Stmn1*^+^, and *Birc5*^+^), and (xi) Tgd (*Trdc*^+^, *Tcrg-c1*^+^, and *Il17a*^+^; Fig. 6A–C). We observed that JHU083-treated B6CaP tumors showed a decreased abundance of FoxP3^+^ CD4_3 clusters [(regulatory T cells (Treg)] and an increase in both stem cell–like *Tcf*^+^ and *Lef1*^+^ CD4_1 and CD_8 T cells (Fig. 6A–D). These functional changes in the CD8 and CD4 TILs were orthogonally validated by flow cytometry of both B6CaP and MB49 tumors, and we identified an increased percentage of stem-like CD8^+^ T cells (CD44^−^CD62L^+^ of CD8^+^) and a decreased percentage of Tregs (FoxP3^+^ of CD4^+^) in response to JHU083 treatment (Fig. 6E).

Next, we investigated whether the stark reprogramming of TAMs in the urologic tumor models in response to JHU083 therapy could relieve immune suppression on T cells and contribute towards an inflammatory immune response in the TME. Autologous CD3/CD28-stimulated CD8^+^-enriched human T cells cocultured with donor-matched M2 macrophages inhibited CD8^+^ T cell proliferation when compared to those cocultured with M1 macrophages, which confirms the immunosuppressive features of M2 macrophages. In contrast, CD8^+^ T cells cocultured with DON-pretreated (0.5 µmol/L) M2 macrophages exhibited enhanced proliferation compared to untreated controls (Fig. 6F and G). Moreover, CD8^+^ T cells cocultured with these reprogrammed M2 macrophages showed increased expression of two key T cell cytokines, TNF and IFNγ, indicating that they were polyfunctional (Fig. 6H). Since DON-pretreated TAMs increased polyfunctional CD8^+^ T cells and stem cell–like CD8^+^ T cells and decreased the proportion of suppressive Tregs, we sought to determine whether ICB therapy in combination with JHU083 would further synergize the antitumor efficacy of JHU083 in urologic tumors. To this end, we treated MB49 tumors with a combination therapy (JHU083 + anti-PD1). Although JHU083-induced increased effector functions of CD8^+^ T cells *in vivo* and *in vitro*, we were unable to demonstrate a superior statistical combinatorial effect of anti-PD1 and JHU083 treatment (Fig. 6I). This is likely due to either the extremely high TGI induced by JHU083 monotherapy or the limitation of tumor volume measurements. It is possible that a reduction in JHU083 treatment concentration would help elucidate the additive/synergistic benefits of combination therapy with anti-PD1.

Overall, our induction of the stem cell–like phenotype of TILs in prostate and bladder cancer tumors is congruent with what has been previously reported (27). In addition to the TME-reprogramming effects on myeloid cells, we also observed a reduction in Tregs in JHU083-treated tumors. Additionally, glutamine blockade of macrophages *in vitro* resulted in increased T cell polyfunctionality and overall relief of immune suppression. While we failed to quantify any superior therapeutic benefit of a combination therapy of JHU083 with anti-PD1 in either of the urologic tumor models, it is plausible that a different combinatorial T cell checkpoint therapy may be beneficial. The studies described here demonstrate the potential value of targeting glutamine metabolism in macrophage/myeloid-rich immunologically challenging urologic tumor models. Additionally, we highlight how glutamine antagonism with JHU083 modulates TAMs, TIMs, tumor cells, and T cells to promote robust antitumor immunity in the TME (Supplementary Fig. S6).

## Discussion

Immunosuppressive TAMs are abundant in most solid tumors and represent a major mechanism of resistance to successful clinical immunotherapeutic efforts. Current clinical attempts to target TAMs therapeutically have had limited success. Studies have shown that both tumor cells (27) and TAMs (59, 60) are metabolically dependent on glutamine (glutamine-addicted), offering a conserved potential single point of failure for these cells. To address this critical need for new immunotherapies and to capitalize on the potential conserved vulnerability of cancer cells and immunosuppressive TAMs, we utilized JHU083, a prodrug variant of DON. JHU083 is a glutamine mimetic with broad glutamine metabolism antagonistic properties. We tested JHU083 across three immunologically myeloid-enriched (“cold”) preclinical models of urological cancers and showed that JHU083 has at least two distinct mechanisms of action in the models tested. First, using scRNA-seq, Western blotting, and metabolic tracing, we showed that JHU083 had a direct antitumor effect on tumor cells and induced apoptosis after a global metabolic shutdown, a reduction in HIF-1α expression levels, and decreased phosphorylation of c-Myc. Second, using bulk and scRNA-seq datasets as well as the AT models of pretreated TAMs and TIMs, we showed that JHU083 reprogrammed immunosuppressive TAMs toward an inflammatory and tumoricidal phenotype characterized by TNFα signaling and durable antitumor effector function. These findings were confirmed orthogonally through flow cytometric detection of increased TNFα production, increased *in vitro* and *in vivo* phagocytosis of labeled tumor cells, increased *Myo1e* expression, decreased intratumoral vasculature, *in vitro* diminished angiogenesis, and alteration of key metabolites. Specifically, we showed that TAMs have increased glycolysis and decreased α-KG levels, indicating a broken TCA cycle more commonly associated with inflammatory M1-macrophages and increased expression of *Sdhb* (succinate dehydrogenase B) and *Il1b* as expected from a broken TCA cycle (55). Furthermore, we showed that T cells play a limited role in the JHU083-mediated antitumor effects seen in these immunologically cold urological cancer models, suggesting that JHU083 may be a strong candidate for cancer indications that are insensitive to ICB therapy. Additionally, we report that JHU083 induced proliferative signaling in several subsets of TAMs and enriched proliferating TAM groups within the TME. RNA velocity predicted that proliferating TAMs were developmentally distinct from infiltrating monocytes, a phenotype of unknown significance in prostate tumors.

Our data are largely in agreement with existing studies utilizing JHU083 in other models that focused on T cells. For example, the AT model using pretreated TAMs and TIMs displays a long-lasting, durable delay in tumor growth within untreated recipients, despite no additional exposure to JHU083 after AT. These data raise questions regarding the length of reprogramming observed in these cells and suggest an epigenetic reprogramming component. Leone and colleagues showed that increased global methylation in T cells following DON treatment causes a memory phenotype (27). We also reported a reduction of α-KG in TAMs after JHU083 treatment, which is known to play a critical role in M2 macrophage polarization through Jmjd3-dependent demethylation of H3K27 in the promoter region of M2 macrophage-specific marker genes (18). Given the decreased levels of α-KG in TAMs due to JHU083-mediated glutamine antagonism, it is possible that this drives the cessation of M2-TAM polarization through epigenetic alteration. Our data also highlight the role of increased TNF production after JHU083 treatment in urological cancer models, whereas Oh and colleagues reported that TNF drives antitumor effects mediated by TAMs in 4T1 tumors following JHU083 therapy (26). Additional reports have also highlighted that increased TNF and inflammatory signaling reprogram TAMs to promote antitumor immunity (61). Regarding the observed increase in phagocytosis, a recent report also postulated a probable direct impact of glutamine metabolism on phagocytosis in TAMs (62). Given the upregulation of *Myo1e* expression, we hypothesize that *Myo1e* plays a key role in the link between glutamine antagonism and phagocytosis. *Myo1e* is important for adhesion turnover during phagocytosis and membrane-cytoskeletal crosstalk for phagocytic cup closure (32) in macrophages.

Clinical trials of therapeutic remodeling via metabolic inhibition of OXPHOS (NCT03291938 and NCT03272256), tryptophan metabolism (NCT02752074), and prostaglandin E2 synthesis (NCT03026140 and NCT03926338) in myeloid cells in various neoplasms have shown promising results (13). However, the failure of the phase III trial of an IDO inhibitor (ECHO301/KN252) highlights the key challenges raised by compensatory expression of similar enzymes in targeting individual metabolic enzymes (63–65). DON acts not only as an irreversible inhibitor of glutamine but also as a mechanism-based inactivator of glutamine-utilizing enzymes affecting multiple pathways (66). Simultaneous inhibition of metabolic pathways based on the differential binding affinities of JHU083 provides a unique opportunity to rule out therapeutic resistance and superior intratumoral delivery (22). In addition to the increased glycolysis and break in the TCA cycle, we showed that targeting multiple enzymes via JHU083 likely impacts purine metabolism in TAMs (67).

Our study provides key insights into the metabolic and phenotypic features of TAMs, TIMs, and tumor cells in urological tumors. Nevertheless, much work remains to be performed, particularly regarding the direct effects of TAMs and TIMs on T cell immunity. Our efforts to combine JHU083 with anti-PD1 immunotherapy in both tumor models to achieve superior antitumor benefits were not entirely successful. This may be due to the limitations of tumor models in representing the true TME. Future experiments to determine the optimal doses of JHU083 and another “checkpoint blockade” target to test combination therapies in urological tumor models are necessary. A key limitation of this study was that we did not examine the long-term effects of JHU083 on tumor metastasis. Instead, we provide a metabolic and phenotypic snapshot of immunosuppressive TAMs in prostate tumors following therapeutic glutamine blockade. This study is unique and valuable because it enriches our understanding of remodeled TAMs following glutamine antagonism using a novel prodrug and provides a basis for the use of JHU083 alone or in combination with existing therapies aimed at targeting TAMs in immunologically cold prostate tumors. JHU083 represents a compelling class of therapeutics aimed at reprogramming rather than the depletion of immunosuppressive TAMs. Our work constitutes an important step forward in promoting novel and effective treatment options for previously immunotherapy-nonresponsive cancers.

## Supplementary Material

Supplementary Legends Supplementary file containing the figure legends for each of the supplemental figures.

Supplementary Table 1 Supplementary Table 1

Supplementary Table 2 Supplementary Table 2

Supplementary Table 3 Supplementary Table 3

Supplementary Figure 1 Supplementary Figure 1

Supplementary Figure 2 Supplementary Figure 2

Supplementary Figure 3 Supplementary Figure 3

Supplementary Figure 4 Supplementary Figure 4

Supplementary Figure 5 Supplementary Figure 5

Supplementary Figure 6 Supplementary Figure 6

## References

[1] Mantovani A , Sozzani S, Locati M, Allavena P, Sica A. Macrophage polarization: tumor-associated macrophages as a paradigm for polarized M2 mononuclear phagocytes. Trends Immunol 2002;23:549–55. 12401408 10.1016/s1471-4906(02)02302-5

[2] Vitale I , Manic G, Coussens LM, Kroemer G, Galluzzi L. Macrophages and metabolism in the tumor microenvironment. Cell Metab 2019;30:36–50. 31269428 10.1016/j.cmet.2019.06.001

[3] Mantovani A , Allavena P, Marchesi F, Garlanda C. Macrophages as tools and targets in cancer therapy. Nat Rev Drug Discov 2022;21:799–820. 35974096 10.1038/s41573-022-00520-5 PMC9380983

[4] El-Kenawi A , Dominguez-Viqueira W, Liu M, Awasthi S, Abraham-Miranda J, Keske A, . Macrophage-derived cholesterol contributes to therapeutic resistance in prostate cancer. Cancer Res 2021;81:5477–90. 34301759 10.1158/0008-5472.CAN-20-4028 PMC8563406

[5] Cassetta L , Pollard JW. Targeting macrophages: therapeutic approaches in cancer. Nat Rev Drug Discov 2018;17:887–904. 30361552 10.1038/nrd.2018.169

[6] Galsky MD , Arija JAA, Bamias A, Davis ID, De Santis M, Kikuchi E, . Atezolizumab with or without chemotherapy in metastatic urothelial cancer (IMvigor130): a multicentre, randomised, placebo-controlled phase 3 trial. Lancet 2020;395:1547–57. 32416780 10.1016/S0140-6736(20)30230-0

[7] Antonarakis ES , Piulats JM, Gross-Goupil M, Goh J, Ojamaa K, Hoimes CJ, . Pembrolizumab for treatment-refractory metastatic castration-resistant prostate cancer: multicohort, open-label phase II KEYNOTE-199 study. J Clin Oncol 2020;38:395–405. 31774688 10.1200/JCO.19.01638 PMC7186583

[8] Powles T , Eder JP, Fine GD, Braiteh FS, Loriot Y, Cruz C, . MPDL3280A (anti-PD-L1) treatment leads to clinical activity in metastatic bladder cancer. Nature 2014;515:558–62. 25428503 10.1038/nature13904

[9] Siegel RL , Miller KD, Wagle NS, Jemal A. Cancer statistics, 2023. CA Cancer J Clin 2023;73:17–48. 36633525 10.3322/caac.21763

[10] Oh DY , Kwek SS, Raju SS, Li T, McCarthy E, Chow E, . Intratumoral CD4^+^ T cells mediate anti-tumor cytotoxicity in human bladder cancer. Cell 2020;181:1612–25.e13. 32497499 10.1016/j.cell.2020.05.017 PMC7321885

[11] Lo CH , Lynch CC. Multifaceted roles for macrophages in prostate cancer skeletal metastasis. Front Endocrinol (Lausanne) 2018;9:247. 29867776 10.3389/fendo.2018.00247 PMC5968094

[12] Pathria P , Louis TL, Varner JA. Targeting tumor-associated macrophages in cancer. Trends Immunol 2019;40:310–27. 30890304 10.1016/j.it.2019.02.003

[13] Goswami S , Anandhan S, Raychaudhuri D, Sharma P. Myeloid cell-targeted therapies for solid tumours. Nat Rev Immunol 2023;23:106–20. 35697799 10.1038/s41577-022-00737-w

[14] O’Neill LA , Pearce EJ. Immunometabolism governs dendritic cell and macrophage function. J Exp Med 2016;213:15–23. 26694970 10.1084/jem.20151570 PMC4710204

[15] Biswas SK , Allavena P, Mantovani A. Tumor-associated macrophages: functional diversity, clinical significance, and open questions. Semin Immunopathol 2013;35:585–600. 23657835 10.1007/s00281-013-0367-7

[16] Colegio OR , Chu NQ, Szabo AL, Chu T, Rhebergen AM, Jairam V, . Functional polarization of tumour-associated macrophages by tumour-derived lactic acid. Nature 2014;513:559–63. 25043024 10.1038/nature13490 PMC4301845

[17] Choi J , Stradmann-Bellinghausen B, Yakubov E, Savaskan NE, Regnier-Vigouroux A. Glioblastoma cells induce differential glutamatergic gene expressions in human tumor-associated microglia/macrophages and monocyte-derived macrophages. Cancer Biol Ther 2015;16:1205–13. 26047211 10.1080/15384047.2015.1056406 PMC4623498

[18] Liu PS , Wang H, Li X, Chao T, Teav T, Christen S, . α-ketoglutarate orchestrates macrophage activation through metabolic and epigenetic reprogramming. Nat Immunol 2017;18:985–94. 28714978 10.1038/ni.3796

[19] Palmieri EM , Menga A, Martin-Perez R, Quinto A, Riera-Domingo C, De Tullio G, . Pharmacologic or genetic targeting of glutamine synthetase skews macrophages toward an M1-like phenotype and inhibits tumor metastasis. Cell Rep 2017;20:1654–66. 28813676 10.1016/j.celrep.2017.07.054 PMC5575233

[20] Kim GW , Lee DH, Jeon YH, Yoo J, Kim SY, Lee SW, . Glutamine synthetase as a therapeutic target for cancer treatment. Int J Mol Sci 2021;22:1701. 33567690 10.3390/ijms22041701 PMC7915753

[21] Xu L , Yin Y, Li Y, Chen X, Chang Y, Zhang H, . A glutaminase isoform switch drives therapeutic resistance and disease progression of prostate cancer. Proc Natl Acad Sci U S A. 2021;118:e2012748118. 33753479 10.1073/pnas.2012748118 PMC8020804

[22] Lemberg KM , Vornov JJ, Rais R, Slusher BS. We’re not “DON” yet: optimal dosing and prodrug delivery of 6-Diazo-5-oxo-L-norleucine. Mol Cancer Ther 2018;17:1824–32. 30181331 10.1158/1535-7163.MCT-17-1148 PMC6130910

[23] Magill GB , Myers WP, Reilly HC, Putnam RC, Magill JW, Sykes MP, . Pharmacological and initial therapeutic observations on 6-diazo-5-oxo-1-norleucine (DON) in human neoplastic disease. Cancer 1957;10:1138–50. 13489662 10.1002/1097-0142(195711/12)10:6<1138::aid-cncr2820100608>3.0.co;2-k

[24] Rais R , Jancarik A, Tenora L, Nedelcovych M, Alt J, Englert J, . Discovery of 6-Diazo-5-oxo-l-norleucine (DON) prodrugs with enhanced CSF delivery in monkeys: a potential treatment for glioblastoma. J Med Chem 2016;59:8621–33. 27560860 10.1021/acs.jmedchem.6b01069

[25] Yamashita AS , da Costa Rosa M, Stumpo V, Rais R, Slusher BS, Riggins GJ. The glutamine antagonist prodrug JHU-083 slows malignant glioma growth and disrupts mTOR signaling. Neurooncol Adv 2021;3:vdaa149. 33681764 10.1093/noajnl/vdaa149 PMC7920530

[26] Oh MH , Sun IH, Zhao L, Leone RD, Sun IM, Xu W, . Targeting glutamine metabolism enhances tumor-specific immunity by modulating suppressive myeloid cells. J Clin Invest 2020;130:3865–84. 32324593 10.1172/JCI131859 PMC7324212

[27] Leone RD , Zhao L, Englert JM, Sun IM, Oh MH, Sun IH, . Glutamine blockade induces divergent metabolic programs to overcome tumor immune evasion. Science 2019;366:1013–21. 31699883 10.1126/science.aav2588 PMC7023461

[28] McGinnis CS , Murrow LM, Gartner ZJ. DoubletFinder: doublet detection in single-cell RNA sequencing data using artificial nearest neighbors. Cell Syst 2019;8:329–37.e4. 30954475 10.1016/j.cels.2019.03.003 PMC6853612

[29] Aran D , Looney AP, Liu L, Wu E, Fong V, Hsu A, . Reference-based analysis of lung single-cell sequencing reveals a transitional profibrotic macrophage. Nat Immunol 2019;20:163–72. 30643263 10.1038/s41590-018-0276-y PMC6340744

[30] Hao Y , Hao S, Andersen-Nissen E, Mauck WM 3rd, Zheng S, Butler A, . Integrated analysis of multimodal single-cell data. Cell 2021;184:3573–87.e29. 34062119 10.1016/j.cell.2021.04.048 PMC8238499

[31] Korsunsky I , Nathan A, Millard N, Raychaudhuri S. Presto scales Wilcoxon and auROC analyses to millions of observations. bioRxiv May 29 2019;653253. Submitted for publication.

[32] Barger SR , Reilly NS, Shutova MS, Li Q, Maiuri P, Heddleston JM, . Membrane-cytoskeletal crosstalk mediated by myosin-I regulates adhesion turnover during phagocytosis. Nat Commun 2019;10:1249. 30890704 10.1038/s41467-019-09104-1 PMC6425032

[33] Haney MS , Bohlen CJ, Morgens DW, Ousey JA, Barkal AA, Tsui CK, . Identification of phagocytosis regulators using magnetic genome-wide CRISPR screens. Nat Genet 2018;50:1716–27. 30397336 10.1038/s41588-018-0254-1 PMC6719718

[34] Danecek P , Bonfield JK, Liddle J, Marshall J, Ohan V, Pollard MO, . Twelve years of SAMtools and BCFtools. Gigascience 2021;10:giab008. 33590861 10.1093/gigascience/giab008 PMC7931819

[35] La Manno G , Soldatov R, Zeisel A, Braun E, Hochgerner H, Petukhov V, . RNA velocity of single cells. Nature 2018;560:494–8. 30089906 10.1038/s41586-018-0414-6 PMC6130801

[36] Bergen V , Lange M, Peidli S, Wolf FA, Theis FJ. Generalizing RNA velocity to transient cell states through dynamical modeling. Nat Biotechnol 2020;38:1408–14. 32747759 10.1038/s41587-020-0591-3

[37] Barkas N , Petukhov V, Nikolaeva D, Lozinsky Y, Demharter S, Khodosevich K, . Joint analysis of heterogeneous single-cell RNA-seq dataset collections. Nat Methods 2019;16:695–8. 31308548 10.1038/s41592-019-0466-z PMC6684315

[38] Lemberg KM , Zhao L, Wu Y, Veeravalli V, Alt J, Aguilar JMH, . The novel glutamine antagonist prodrug JHU395 has antitumor activity in malignant peripheral nerve sheath tumor. Mol Cancer Ther 2020;19:397–408. 31594823 10.1158/1535-7163.MCT-19-0319 PMC7007868

[39] Alt J , Gori SS, Lemberg KM, Pal A, Veeravalli V, Wu Y, . Glutamine antagonist GA-607 causes a dramatic accumulation of FGAR which can be used to monitor target engagement. Curr Drug Metab 2021;22:735–45. 34488583 10.2174/1389200222666210831125041 PMC8684803

[40] Zarif JC , Hernandez JR, Verdone JE, Campbell SP, Drake CG, Pienta KJ. A phased strategy to differentiate human CD14+monocytes into classically and alternatively activated macrophages and dendritic cells. Biotechniques 2016;61:33–41. 27401672 10.2144/000114435 PMC5504907

[41] Kfoury Y , Baryawno N, Severe N, Mei S, Gustafsson K, Hirz T, . Human prostate cancer bone metastases have an actionable immunosuppressive microenvironment. Cancer Cell 2021;39:1464–78.e8. 34719426 10.1016/j.ccell.2021.09.005 PMC8578470

[42] Simons BW , Kothari V, Benzon B, Ghabili K, Hughes R, Zarif JC, . A mouse model of prostate cancer bone metastasis in a syngeneic immunocompetent host. Oncotarget 2019;10:6845–54. 31839878 10.18632/oncotarget.27317 PMC6901336

[43] White-Gilbertson S , Davis M, Voelkel-Johnson C, Kasman LM. Sex differences in the MB49 syngeneic, murine model of bladder cancer. Bladder (San Franc) 2016;3:e22. 26998503 10.14440/bladder.2016.73 PMC4795170

[44] Thompson TC , Southgate J, Kitchener G, Land H. Multistage carcinogenesis induced by ras and myc oncogenes in a reconstituted organ. Cell 1989;56:917–30. 2538247 10.1016/0092-8674(89)90625-9

[45] Moynihan KD , Opel CF, Szeto GL, Tzeng A, Zhu EF, Engreitz JM, . Eradication of large established tumors in mice by combination immunotherapy that engages innate and adaptive immune responses. Nat Med 2016;22:1402–10. 27775706 10.1038/nm.4200 PMC5209798

[46] Zeisberger SM , Odermatt B, Marty C, Zehnder-Fjallman AH, Ballmer-Hofer K, Schwendener RA. Clodronate-liposome-mediated depletion of tumour-associated macrophages: a new and highly effective antiangiogenic therapy approach. Br J Cancer 2006;95:272–81. 16832418 10.1038/sj.bjc.6603240 PMC2360657

[47] Geissmann F , Manz MG, Jung S, Sieweke MH, Merad M, Ley K. Development of monocytes, macrophages, and dendritic cells. Science 2010;327:656–61. 20133564 10.1126/science.1178331 PMC2887389

[48] Ziegler-Heitbrock L , Ancuta P, Crowe S, Dalod M, Grau V, Hart DN, . Nomenclature of monocytes and dendritic cells in blood. Blood 2010;116:e74-80. 20628149 10.1182/blood-2010-02-258558

[49] Vorselen D , Barger SR, Wang Y, Cai W, Theriot JA, Gauthier NC, . Phagocytic “teeth” and myosin-II “jaw” power target constriction during phagocytosis. Elife 2021;10:e68627. 34708690 10.7554/eLife.68627 PMC8585483

[50] Bott AJ , Shen J, Tonelli C, Zhan L, Sivaram N, Jiang YP, . Glutamine anabolism plays a critical role in pancreatic cancer by coupling carbon and nitrogen metabolism. Cell Rep 2019;29:1287–98.e6. 31665640 10.1016/j.celrep.2019.09.056 PMC6886125

[51] Collins SL , Oh MH, Sun IH, Chan-Li Y, Zhao L, Powell JD, . mTORC1 signaling regulates proinflammatory macrophage function and metabolism. J Immunol 2021;207:913–22. 34290107 10.4049/jimmunol.2100230

[52] Geeraerts X , Fernandez-Garcia J, Hartmann FJ, de Goede KE, Martens L, Elkrim Y, . Macrophages are metabolically heterogeneous within the tumor microenvironment. Cell Rep 2021;37:110171. 34965415 10.1016/j.celrep.2021.110171

[53] Sheldon RD , Ma EH, DeCamp LM, Williams KS, Jones RG. Interrogating *in vivo* T-cell metabolism in mice using stable isotope labeling metabolomics and rapid cell sorting. Nat Protoc 2021;16:4494–521. 34349284 10.1038/s41596-021-00586-2

[54] Li S , Yu J, Huber A, Kryczek I, Wang Z, Jiang L, . Metabolism drives macrophage heterogeneity in the tumor microenvironment. Cell Rep 2022;39:110609. 35385733 10.1016/j.celrep.2022.110609 PMC9052943

[55] Mills EL , Kelly B, Logan A, Costa ASH, Varma M, Bryant CE, . Succinate dehydrogenase supports metabolic repurposing of mitochondria to drive inflammatory macrophages. Cell 2016;167:457–70.e13. 27667687 10.1016/j.cell.2016.08.064 PMC5863951

[56] Tannahill GM , Curtis AM, Adamik J, Palsson-McDermott EM, McGettrick AF, Goel G, . Succinate is an inflammatory signal that induces IL-1β through HIF-1α. Nature 2013;496:238–42. 23535595 10.1038/nature11986 PMC4031686

[57] Yoo HC , Yu YC, Sung Y, Han JM. Glutamine reliance in cell metabolism. Exp Mol Med 2020;52:1496–516. 32943735 10.1038/s12276-020-00504-8 PMC8080614

[58] Schcolnik-Cabrera A , Chavez-Blanco A, Dominguez-Gomez G, Juarez M, Vargas-Castillo A, Ponce-Toledo RI, . Pharmacological inhibition of tumor anabolism and host catabolism as a cancer therapy. Sci Rep 2021;11:5222. 33664364 10.1038/s41598-021-84538-6 PMC7933231

[59] Mukha A , Kahya U, Linge A, Chen O, Lock S, Lukiyanchuk V, . GLS-driven glutamine catabolism contributes to prostate cancer radiosensitivity by regulating the redox state, stemness and ATG5-mediated autophagy. Theranostics 2021;11:7844–68. 34335968 10.7150/thno.58655 PMC8315064

[60] Reinfeld BI , Madden MZ, Wolf MM, Chytil A, Bader JE, Patterson AR, . Cell-programmed nutrient partitioning in the tumour microenvironment. Nature 2021;593:282–8. 33828302 10.1038/s41586-021-03442-1 PMC8122068

[61] Sarode P , Zheng X, Giotopoulou GA, Weigert A, Kuenne C, Gunther S, . Reprogramming of tumor-associated macrophages by targeting β-catenin/FOSL2/ARID5A signaling: a potential treatment of lung cancer. Sci Adv 2020;6:eaaz6105. 32548260 10.1126/sciadv.aaz6105 PMC7274802

[62] Li J , Ye Y, Liu Z, Zhang G, Dai H, Li J, . Macrophage mitochondrial fission improves cancer cell phagocytosis induced by therapeutic antibodies and is impaired by glutamine competition. Nat Cancer 2022;3:453–70. 35484420 10.1038/s43018-022-00354-5

[63] Muller AJ , Manfredi MG, Zakharia Y, Prendergast GC. Inhibiting IDO pathways to treat cancer: lessons from the ECHO-301 trial and beyond. Semin Immunopathol 2019;41:41–8. 30203227 10.1007/s00281-018-0702-0

[64] Long GV , Dummer R, Hamid O, Gajewski TF, Caglevic C, Dalle S, . Epacadostat plus pembrolizumab versus placebo plus pembrolizumab in patients with unresectable or metastatic melanoma (ECHO-301/KEYNOTE-252): a phase 3, randomised, double-blind study. Lancet Oncol 2019;20:1083–97. 31221619 10.1016/S1470-2045(19)30274-8

[65] Labadie BW , Bao R, Luke JJ. Reimagining IDO pathway inhibition in cancer immunotherapy via downstream focus on the tryptophan-kynurenine-aryl hydrocarbon axis. Clin Cancer Res 2019;25:1462–71. 30377198 10.1158/1078-0432.CCR-18-2882 PMC6397695

[66] Rais R , Lemberg KM, Tenora L, Arwood ML, Pal A, Alt J, . Discovery of DRP-104, a tumor-targeted metabolic inhibitor prodrug. Sci Adv 2022;8:eabq5925. 36383674 10.1126/sciadv.abq5925 PMC9668306

[67] Yokoyama Y , Estok TM, Wild R. Sirpiglenastat (DRP-104) induces antitumor efficacy through direct, broad antagonism of glutamine metabolism and stimulation of the innate and adaptive immune systems. Mol Cancer Ther 2022;21:1561–72. 35930753 10.1158/1535-7163.MCT-22-0282

