# Supplementary material for: Metabolic Reprogramming of Tumor-Associated Macrophages Using Glutamine Antagonist JHU083 Drives Tumor Immunity in Myeloid-Rich Prostate and Bladder Cancers
Source: Cancer Immunol Res. 2024 Apr 26;12(7):854–75. doi: 10.1158/2326-6066.CIR-23-1105 (PMC11217738; doi:10.1158/2326-6066.CIR-23-1105)
Supplement: Supplementary Legends — Supplementary file containing the figure legends for each of the supplemental figures. [file cir-23-1105_supplementary_legends_suppsm.docx]

**
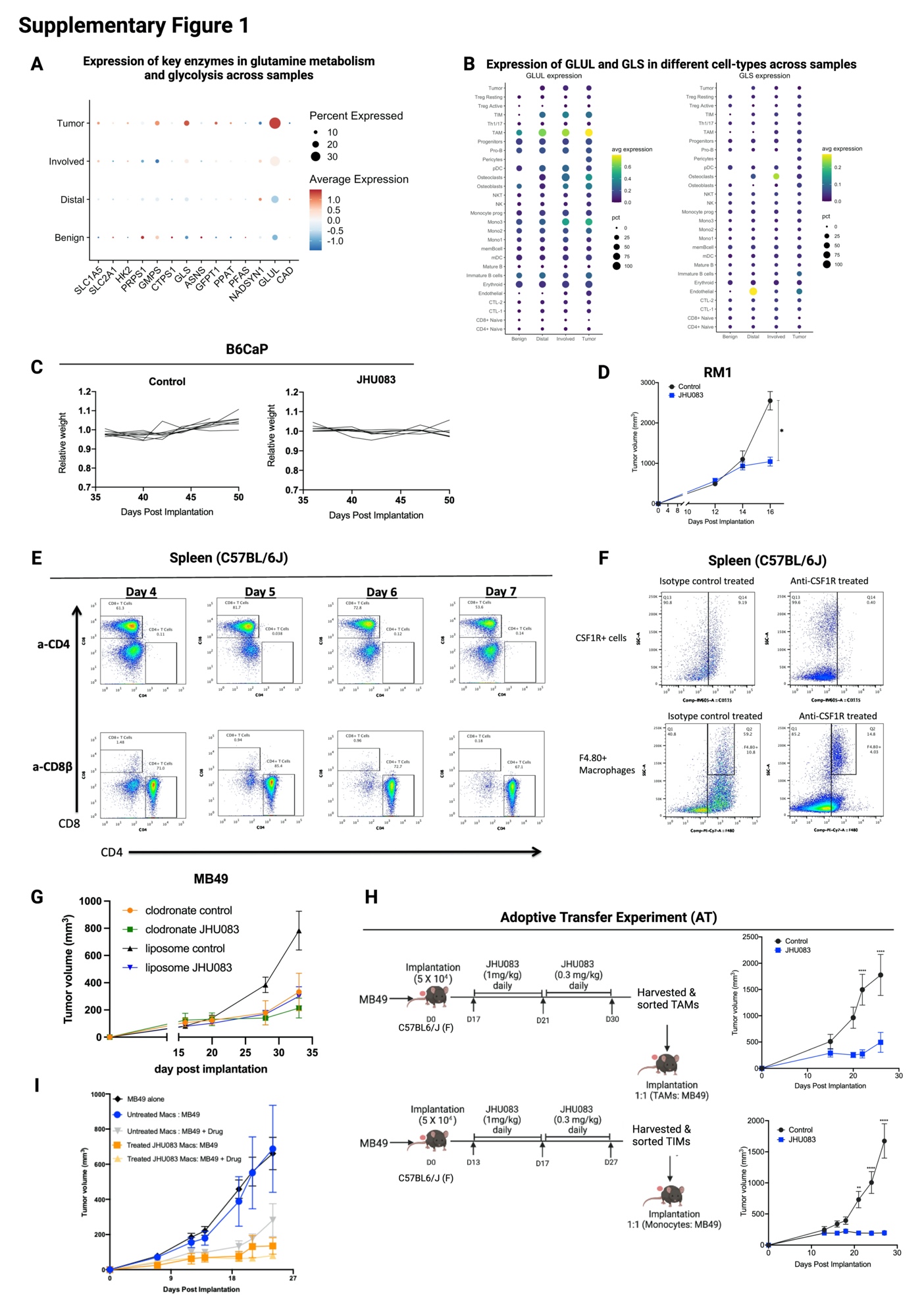
**

**Supplementary Figure 1. (A)** Dot plot showing expression of key enzymes in glutamine metabolism and glycolysis and fractional abundance of samples (benign, distal, involved, and tumor) expressing enzymes. **(B)** Dot plots showing expression of *GLUL* and *GLS* and fractional abundance of different cell types expressing these enzymes in different patient samples (benign, distal, involved, and tumor). Data for figure **A** and **B** are derived from the previously published and publicly available single-cell RNA sequencing (scRNAseq) integrated dataset from 9 metastatic prostate cancer bone tumors, and 7 benign BM control patient samples by Kfoury *et al*. 2021. **(C)** Body weights of vehicle-treated and JHU083-treated mice bearing B6CaP tumors across the therapeutic window.**(D)** Anti-tumor activity of glutamine-antagonist prodrug JHU083 in RM-1 PCa tumors (n=3/ group). **(E)** Confirmation of anti-CD4 and anti-CD8β antibody-mediated T cell depletion in the spleen following intraperitoneal (i.p.) injections at 200 μg antibodies per treatment in C57BL/6J mice, and **(F)** Optimization and testing of anti-CSF1R mediated macrophage depletion in the spleen following intraperitoneal (i.p.) injections at 300 μg antibodies in C57BL/6J mice on Day 3. **(G)** tumor volume measurement of MB49 bearing mice (n=3-6) treated with either control liposome vehicle or clodronate encapsulated in liposome vehicle for macrophage depletion. **(H)** Schematic representation for adoptive transfer experiments (ADT) of JHU083-treated TAMs (n=7 or 8/group) and TIMs (n=13/group) from the MB49 tumor-bearing mice and donor mice. Tumor volume measurements were carried out following ADT. **(I)** JHU083 treatment following adoptive transfer in TAMs (Figure 1J), wherein there are 2 additional groups of drug (JHU083) treatment post ADT. Statistical analyses were done with either t-test or two-way ANOVA using Bonferroni's multiple comparisons (**P* < 0.05, ***P* < 0.01, ****P* < 0.001, *****P* < 0.0001).

**


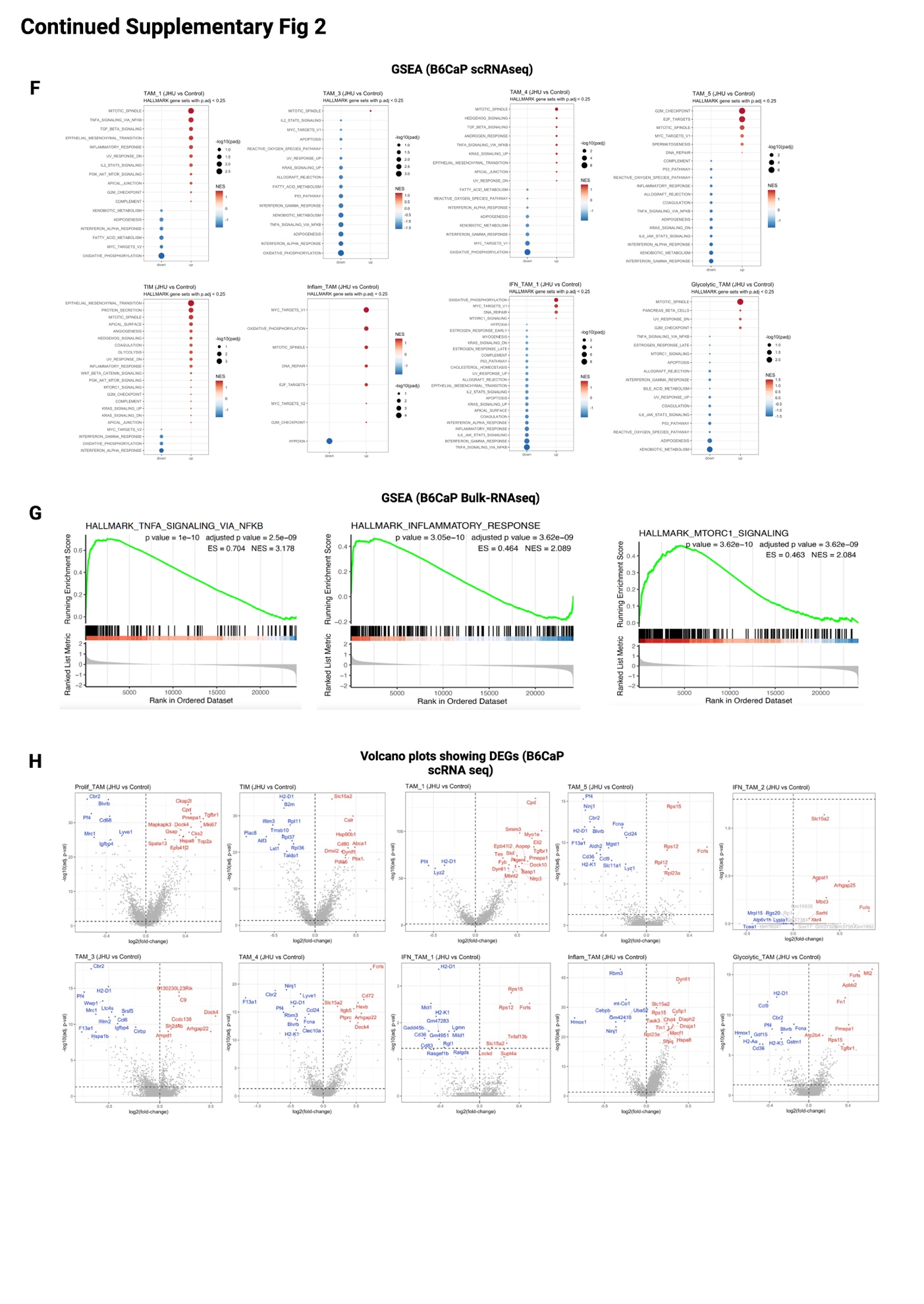

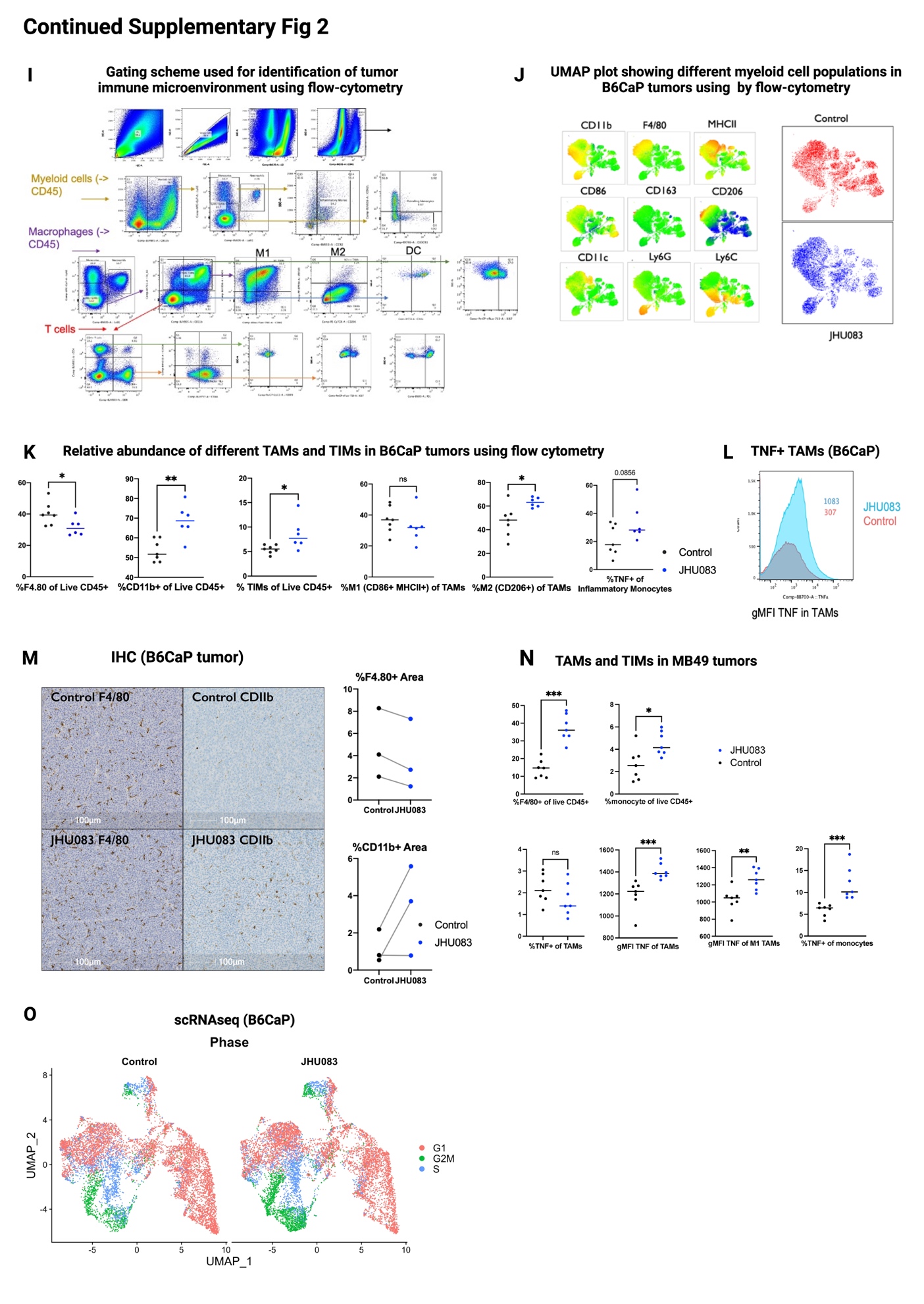
**

**Supplementary Figure 2. (A)** Tumor volume measurement of B6CaP tumors at an early time point following JHU083 treatment. Briefly, control and JHU083-treated tumors were used for scRNAseq (Day 7 post-treatment) (n=6) on enriched live CD45^+^ and CD45^-^ cells. **(B)** Tumor volume measurement of B6CaP tumors following JHU treatment. Tumors at a late time point (Day 18 post-treatment) (n=6/ group) were used for isolation of FACS-sorted TAMs (Live CD45^+^ CD3^-^ Ly6G^-^ CD11b^+^ F4.80^+^) for bulk RNAseq. The diagram on the right-hand side shows the PCA analysis. **(C)** UMAP plots showing different immune cell clusters within the CD45^+^ compartment following scRNAseq (left) and dot plot showing expression levels of marker genes identified by SingleR algorithm to confirm cell-type identities (right) across all samples. **(D)** UMAP plots showing identification and relative abundance of different immune cells within CD45^+^ compartments compared between JHU083-treated cells vs. control samples (left). Density plot (UMAP) showing the differential abundance of JHU083-treated vs. control samples (right). **(E)** Dot plot showing highly expressed marker genes within each TAM/TIM cluster identified within the parent macrophage/monocyte cluster (*Adgre1^+^ Mrc1^+^Itgam^+^Ccr2^+^*). **(F)** Dot plots showing relative enrichment of different hallmark pathways using GSEA (data obtained from bulk RNAseq of TAMs in JHU083-treated vs. control) in each cluster of the macrophage/monocyte subset. **(G)** GSEA enrichment plots for the Hallmark TNFA_signaling_via_NfKb, Inflammatory_response, and mTORC1_signaling gene sets from bulk RNA-sequencing of TAMs (JHU083-treated vs. control). **(H)** Volcano plots representing top DEGs from TAM/TIM clusters identified in scRNAseq (JHU083-treated vs. control). **(I)** Gating schematic utilized for flow cytometry-based analyses of the tumor microenvironment (TME) in MB49 and B6CaP tumors based on cell-surface and intracellular expression of phenotypic markers. **(J)** UMAP plots showing different myeloid cell populations and their relative abundance in B6CaP tumors using flow cytometry analyses in JHU083-treated vs. control tumors. **(K)** Differential abundance of different TAM and TIM subsets in B6CaP tumors (control vs. JHU083-treated) based on cell-surface and intracellular expression of different phenotypic markers using flow cytometry. **(L)** Geometric mean of TNF expression of a representative TAM population in control vs. JHU083-treated tumors. **(M)** Representative immunohistochemistry (IHC) images and quantification of F4/80 and CD11b in three independent JHU083 treated B6CaP tumors experiments (IHC was performed in a single tumor randomly selected from each experiment). **(N)** Differential abundance of TAMs and TIMs, percentage of TNF^+^ TAMs and TIMs, also gMFI of TNF in TAMs in MB49 tumors based on cell-surface and intracellular expression of phenotypic markers using flow cytometry. **(O)** UMAP plots of the macrophage/monocyte subset and their predicted cell cycle phases. DEGs were calculated with DESeq2 in the bulk RNA-seq data, and with the Wilcoxon rank-sum test for the scRNA-seq data. Statistical analyses done with either t-test or two-way ANOVA using Bonferroni's multiple comparisons (**P* < 0.05, ***P* < 0.01, ****P* < 0.001, *****P* < 0.0001).

**
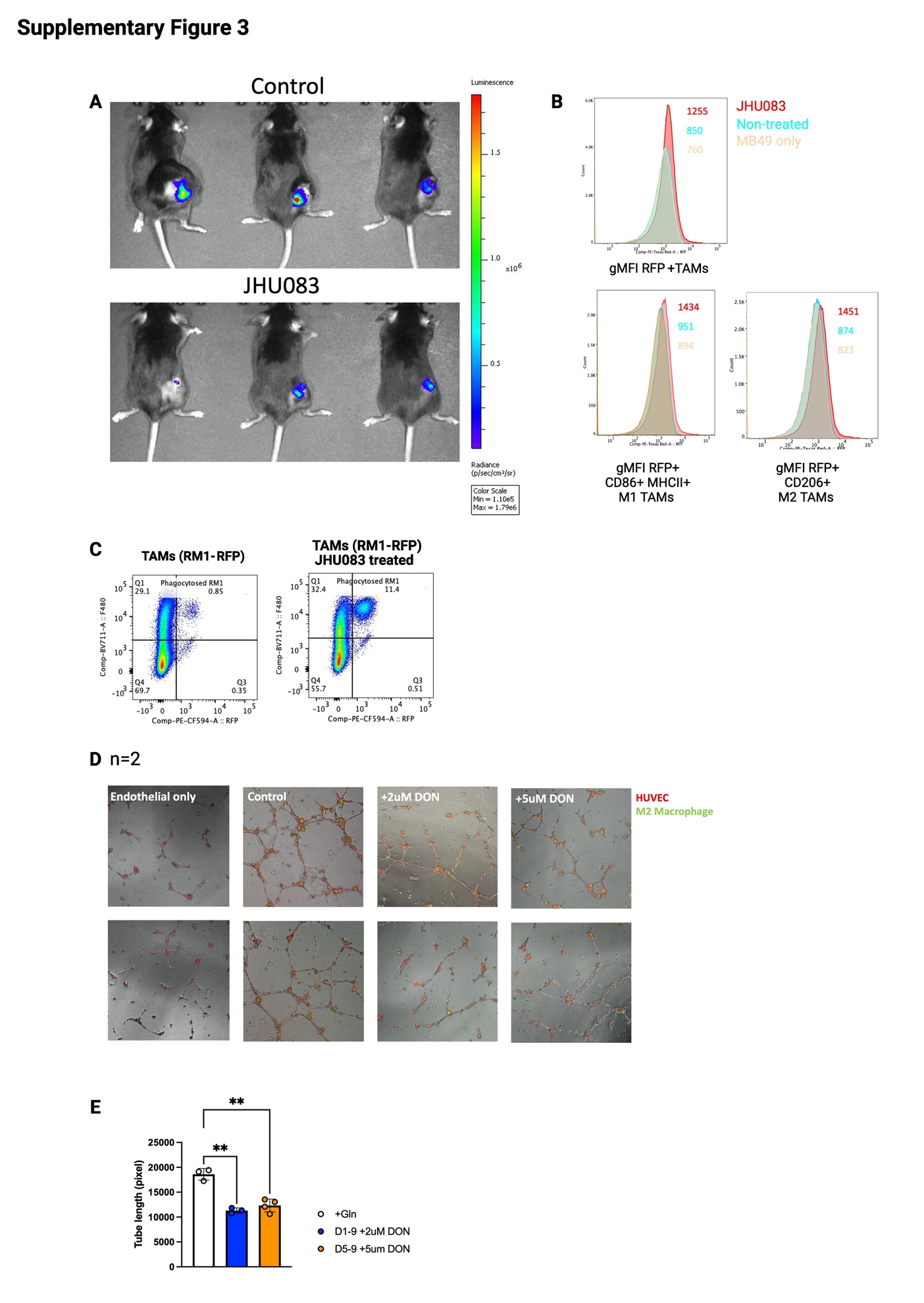
**

**Supplementary Figure 3.** **(A)** IVIS-based quantification of tumor growth by luciferase bioluminescence activity in MB49-RFP^+^ tumors. **(B)** RFP gMFI expression in TAMs, M1 TAMs, and M2 TAMs and **(C)** Representative flow cytometry plots of RFP^+^ RM1 tumor TAMs. (**D & E**) *In vitro* endothelial assay. Briefly, either non treated or DON-treated, PBMC-derived macrophages were co-cultured with untreated endothelial HUVEC2 cells on solidified Matrigel, and endothelial tube length quantified using ImageJ (n=2 experiments).

**
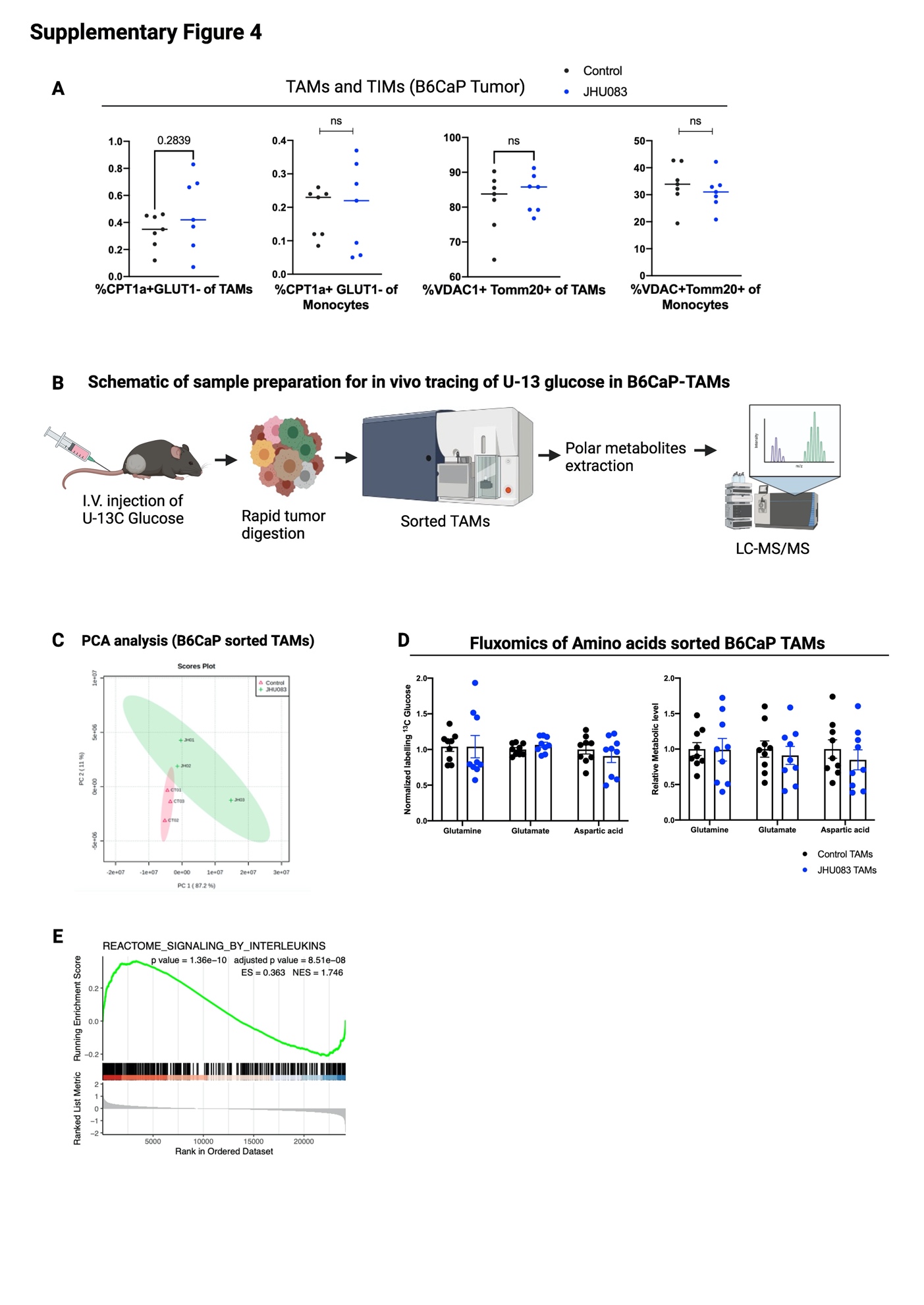
**

**Supplementary Figure 4. Metabolic reprogramming of TAMs after JHU083 treatment.** **(A)** Percentage of CPT1α^+^ GLUT^-^, VDAC1 TOMM20^+^ cells in TAMs and TIMs in B6CaP tumors as determined using flow cytometric analysis **(B)** Schematic diagram showing sample preparation for *in vivo* tracing of U-^13^C glucose in B6CaP TAMs after rapid digestion and FACS sorting. **(C)** PCA analysis of TAMs (B6CaP) for targeted metabolite analysis using LC-MS/MS (n=3/group). **(D)** Normalized relative labeled metabolites and their abundances from U-^13^C glucose in amino acids in TAMs derived from B6CaP tumors (n=9/group from 2 independent experiments), and **(E)** GSEA showing pathway enrichment for the Reactome Signaling by Interleukins from DEGs identified in bulk-RNA seq data in B6CaP sorted TAMs. Statistical analyses were done with unpaired t-test. (**P* < 0.05, ***P* < 0.01, ****P* < 0.001, *****P* < 0.0001).

**
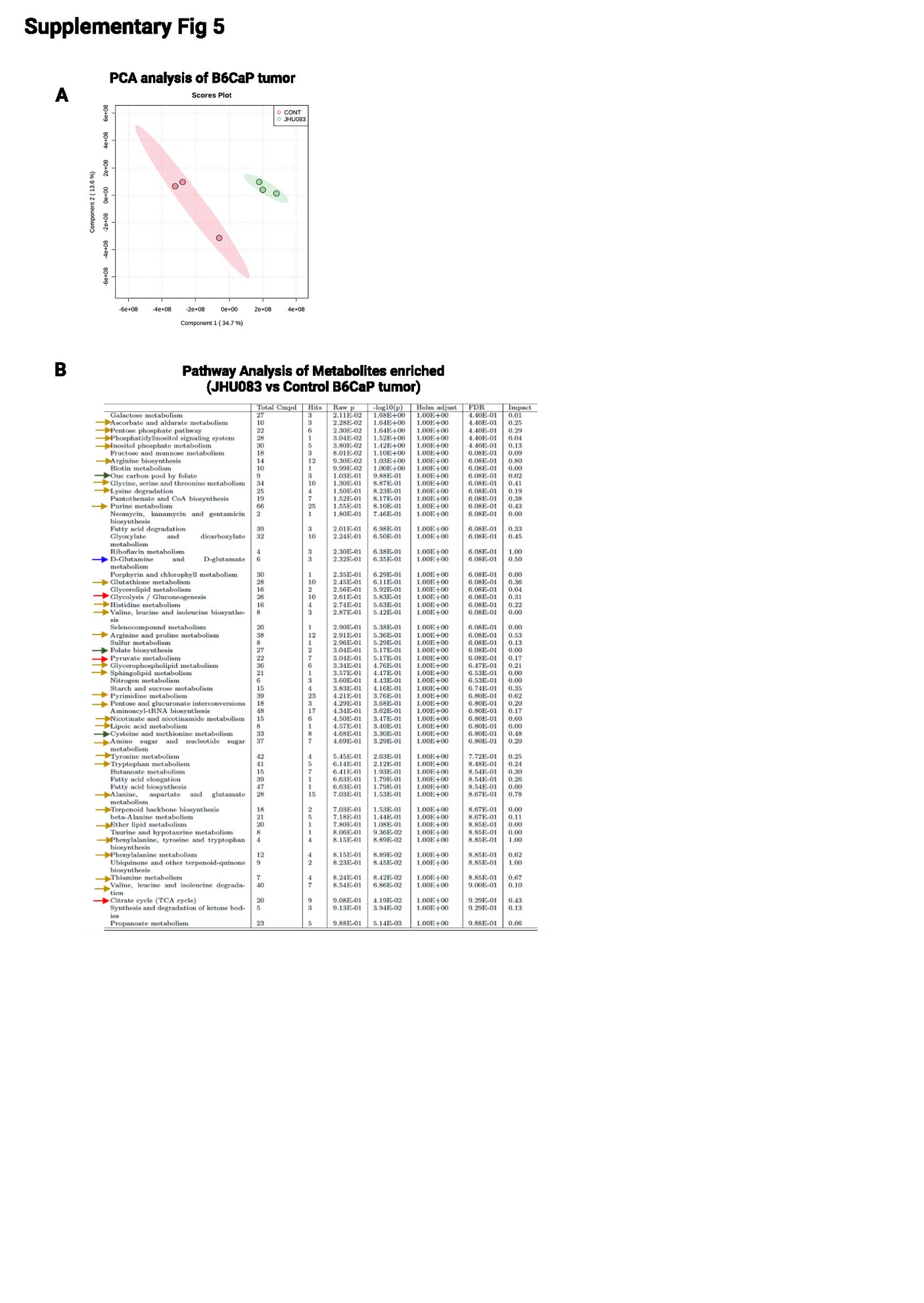
**

**Supplementary Figure 5.** **(A)** PCA analysis of B6CaP tumor samples submitted for LC/MS-MS analysis, and **(B)** Pathway analysis of differential metabolites upregulated by JHU083-treated B6CaP tumors.

**
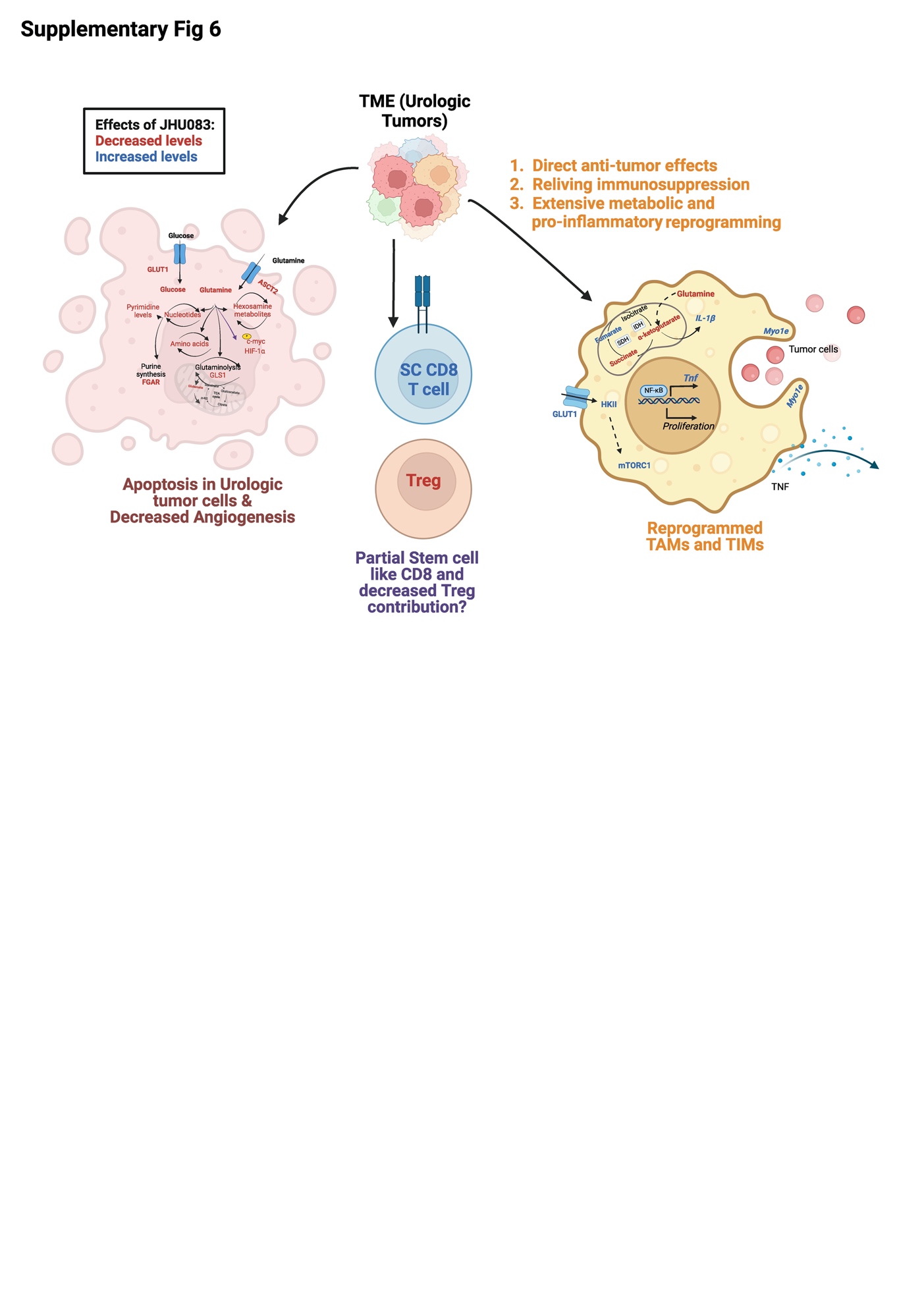
**

**Supplementary Figure 6 (supporting data for figure 6). (A)** Schematic cartoon diagram representation of the proposed model of anti-tumor immunity driven by glutamine inhibition induced by JHU083 in urologic tumors.
