# Supplementary material for: Metabolic Reprogramming of Tumor-Associated Macrophages Using Glutamine Antagonist JHU083 Drives Tumor Immunity in Myeloid-Rich Prostate and Bladder Cancers
Source: Cancer Immunol Res. 2024 Apr 26;12(7):854–75. doi: 10.1158/2326-6066.CIR-23-1105 (PMC11217738; doi:10.1158/2326-6066.CIR-23-1105)
Supplement: Supplementary Table 1 [file cir-23-1105_supplementary_table_1_suppst1.docx]

**Supplementary Table 1: Antibodies used in flow cytometry experiments and FACs sorting.**

| Marker | Fluorophore | Vendor | Cat # |
| --- | --- | --- | --- |
| CD206 | PE CF594 | BD | 141732 |
| CD86 | Pe/Cy5 | Biolegend | 105016 |
| CD62L | PECy7 | BD | 560516 |
| CD11b | AF700 | Biolegend | 101222 |
| CD4 | APC Cy7 | BD | 565650 |
| Ly6G | efluor 450 | Thermo | 48-5931-82 |
| CD44 | BV480 | BD | 566200 |
| TCRb | BV510 | BD | 563221 |
| CD45 | Pacific Orange | Invitrogen | MCD4530 |
| Ly6C | BV570 | Biolegend | 128030 |
| PD-1 | BV605 | Biolegend | 135220 |
| CD8 | BV650 | Biolegend | 100742 |
| MHCII | BV711 | Biolegend | 107643 |
| CD11c | BV750 | Biolegend | 117357 |
| F4/80 | BV785 | Biolegend | 123141 |
| CD163 | PE | Thermofisher | 12-1631-82 |
| CPT1a | AF488 | Abcam | ab171449 |
| VDAC1 | AF532 | Abcam | ab14734 |
| FOXP3 | PerCpCy5.5 | Thermo | 45-5773-82 |
| Ki67 | PerCp-eFluor710 | Thermo | 46-5698-82 |
| GLUT1 | AF647 | Abcam | ab195020 |
| Hexokinase II | - | Abcam | ab228819 |
| DyLight® 680 conjugation kit (Fast) |  | Abcam | ab201804 |
| Tomm20 | AF405 | Abcam | ab210047 |
| CD45 | V510 | Biolegend | 103138 |
| CD11b | UV805 | BD | 741934 |
| F4/80 | V711 | BD | 565612 |
| MHCII | UV395 | BD | 743876 |
| CX3CRI | V786 | Biolegend | 149029 |
| CD206 | PE-Cy7 | Biolegend | 141720 |
| CD163 | PE Dazzle | Biolegend | 155316 |
| CD86 | APCR700 | BD | 565479 |
| Ly6C | APC-fire 750 | Biolegend | 128046 |
| Ly6G | V605 | Biolegend | 127639 |
| CCR2 | BV650 | Biolegend | 150613 |
| L/D | V575 | BD | 565694 |
| GLUT1 | AF647 | Abcam | ab195020 |
| CD4 | BUV661 | BD | 612974 |
| CD8 | BUV563 | BD | 748535 |
| CD44 | BUV737 | BD | 612799 |
| CD62L | BUV615 | BD | 752311 |
| TNF | BB700 | BD | 566510 |
| Arginase | PE | Thermofisher | 12-3697-82 |
| CPT1a | FITC | Abcam | ab171449 |
| CD31 | AF594 | Biolegend | 303126 |
| CD11b | FITC | Biolegend | 101206 |
| F4/80 | PeCy7 | Biolegend | 123114 |
| CD3 | BV786 | BD | 564010 |
| Ly6G | BV421 | Biolegend | 127628 |
| Ly6C | AF700 | Biolegend | 128024 |
