# Supplementary material for: Metabolic Reprogramming of Tumor-Associated Macrophages Using Glutamine Antagonist JHU083 Drives Tumor Immunity in Myeloid-Rich Prostate and Bladder Cancers
Source: Cancer Immunol Res. 2024 Apr 26;12(7):854–75. doi: 10.1158/2326-6066.CIR-23-1105 (PMC11217738; doi:10.1158/2326-6066.CIR-23-1105)
Supplement: Supplementary Table 2 [file cir-23-1105_supplementary_table_2_suppst2.docx]

**Supplementary Table 2. Details of IHC antibodies used**

| **Antibody** | **Vendor** | **# Catalog** | **Titration/Dilution** |
| --- | --- | --- | --- |
| CD31 | AbCam | ab182981 | 1:1000 |
| CD11b | AbCam | ab133357 | 1:8000 |
| F4/80 | Bio-Rad | MCA497 | 1:2000 |
