# Supplementary material for: Metabolic Reprogramming of Tumor-Associated Macrophages Using Glutamine Antagonist JHU083 Drives Tumor Immunity in Myeloid-Rich Prostate and Bladder Cancers
Source: Cancer Immunol Res. 2024 Apr 26;12(7):854–75. doi: 10.1158/2326-6066.CIR-23-1105 (PMC11217738; doi:10.1158/2326-6066.CIR-23-1105)
Supplement: Supplementary Table 3 [file cir-23-1105_supplementary_table_3_suppst3.docx]

**Supplementary Table 3. Details of Western Blot antibodies used**

| **Antibody** | **Vendor** | **# Cat** | **Dilution** |
| --- | --- | --- | --- |
| ASCT2 | Cell Signaling Technology | 5345S | 1:1000 |
| KAG | Proteintech | 20170-1-AP | 1:500 |
| GAC | Proteintech | 19958-1-AP | 1:500 |
| GLS2 | Abcam | ab113509 | 1:1000 |
| GLUT1 | Abcam | ab195020 | 1:1000 |
| GLUL | Cell Signaling Technology | 80636 | 1:1000 |
| HIF1alpha | Cell Signaling Technology | 14179s | 1:500 |
| Cleaved Caspase-3 | Cell Signaling Technology | 9661s | 1:1000 |
| c-Myc | Abcam | ab32072 | 1:1000 |
| c-Myc S62 | Abcam | ab51156 | 1:1000 |
| c-Myc T58 | Abcam | ab28842 | 1:1000 |
| β-actin | Cell Signaling Technology | 4967L | 1:1000 |
