# Supplementary material for: Metabolic Reprogramming of Tumor-Associated Macrophages Using Glutamine Antagonist JHU083 Drives Tumor Immunity in Myeloid-Rich Prostate and Bladder Cancers
Source: Cancer Immunol Res. 2024 Apr 26;12(7):854–75. doi: 10.1158/2326-6066.CIR-23-1105 (PMC11217738; doi:10.1158/2326-6066.CIR-23-1105)
Supplement: Supplementary Figure 1 [file cir-23-1105_supplementary_figure_1_suppsf1.docx]

**
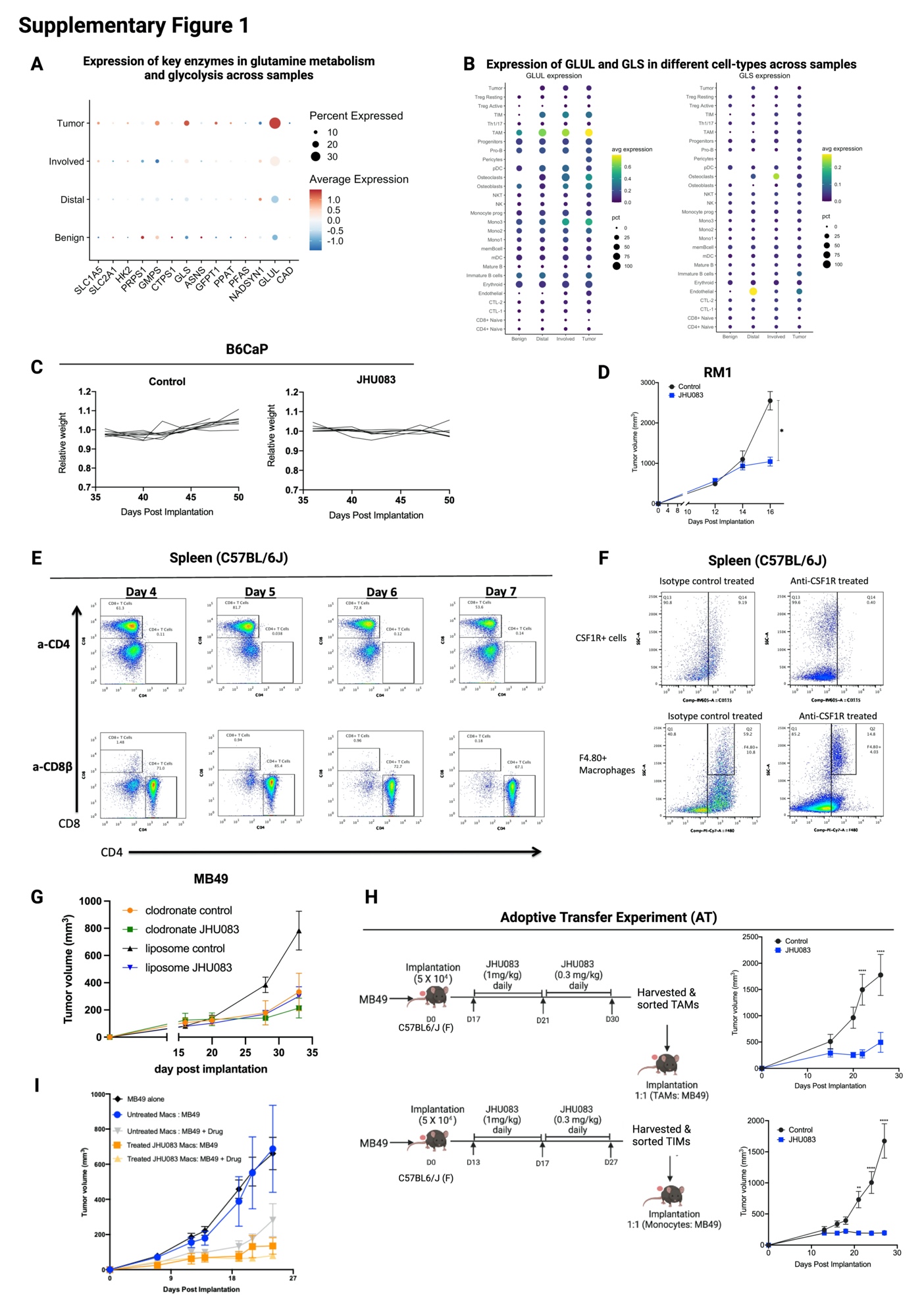
**

**Supplementary Figure 1. (A)** Dot plot showing expression of key enzymes in glutamine metabolism and glycolysis and fractional abundance of samples (benign, distal, involved, and tumor) expressing enzymes. **(B)** Dot plots showing expression of *GLUL* and *GLS* and fractional abundance of different cell types expressing these enzymes in different patient samples (benign, distal, involved, and tumor). Data for figure **A** and **B** are derived from the previously published and publicly available single-cell RNA sequencing (scRNAseq) integrated dataset from 9 metastatic prostate cancer bone tumors, and 7 benign BM control patient samples by Kfoury *et al*. 2021. **(C)** Body weights of vehicle-treated and JHU083-treated mice bearing B6CaP tumors across the therapeutic window.**(D)** Anti-tumor activity of glutamine-antagonist prodrug JHU083 in RM-1 PCa tumors (n=3/ group). **(E)** Confirmation of anti-CD4 and anti-CD8β antibody-mediated T cell depletion in the spleen following intraperitoneal (i.p.) injections at 200 μg antibodies per treatment in C57BL/6J mice, and **(F)** Optimization and testing of anti-CSF1R mediated macrophage depletion in the spleen following intraperitoneal (i.p.) injections at 300 μg antibodies in C57BL/6J mice on Day 3. **(G)** tumor volume measurement of MB49 bearing mice (n=3-6) treated with either control liposome vehicle or clodronate encapsulated in liposome vehicle for macrophage depletion. **(H)** Schematic representation for adoptive transfer experiments (ADT) of JHU083-treated TAMs (n=7 or 8/group) and TIMs (n=13/group) from the MB49 tumor-bearing mice and donor mice. Tumor volume measurements were carried out following ADT. **(I)** JHU083 treatment following adoptive transfer in TAMs (Figure 1J), wherein there are 2 additional groups of JHU083 treatment post ADT. Statistical analyses were done with either t-test or two-way ANOVA using Bonferroni's multiple comparisons (**P* < 0.05, ***P* < 0.01, ****P* < 0.001, *****P* < 0.0001).
