# Supplementary material for: Metabolic Reprogramming of Tumor-Associated Macrophages Using Glutamine Antagonist JHU083 Drives Tumor Immunity in Myeloid-Rich Prostate and Bladder Cancers
Source: Cancer Immunol Res. 2024 Apr 26;12(7):854–75. doi: 10.1158/2326-6066.CIR-23-1105 (PMC11217738; doi:10.1158/2326-6066.CIR-23-1105)
Supplement: Supplementary Figure 2 [file cir-23-1105_supplementary_figure_2_suppsf2.docx]

**

**

**
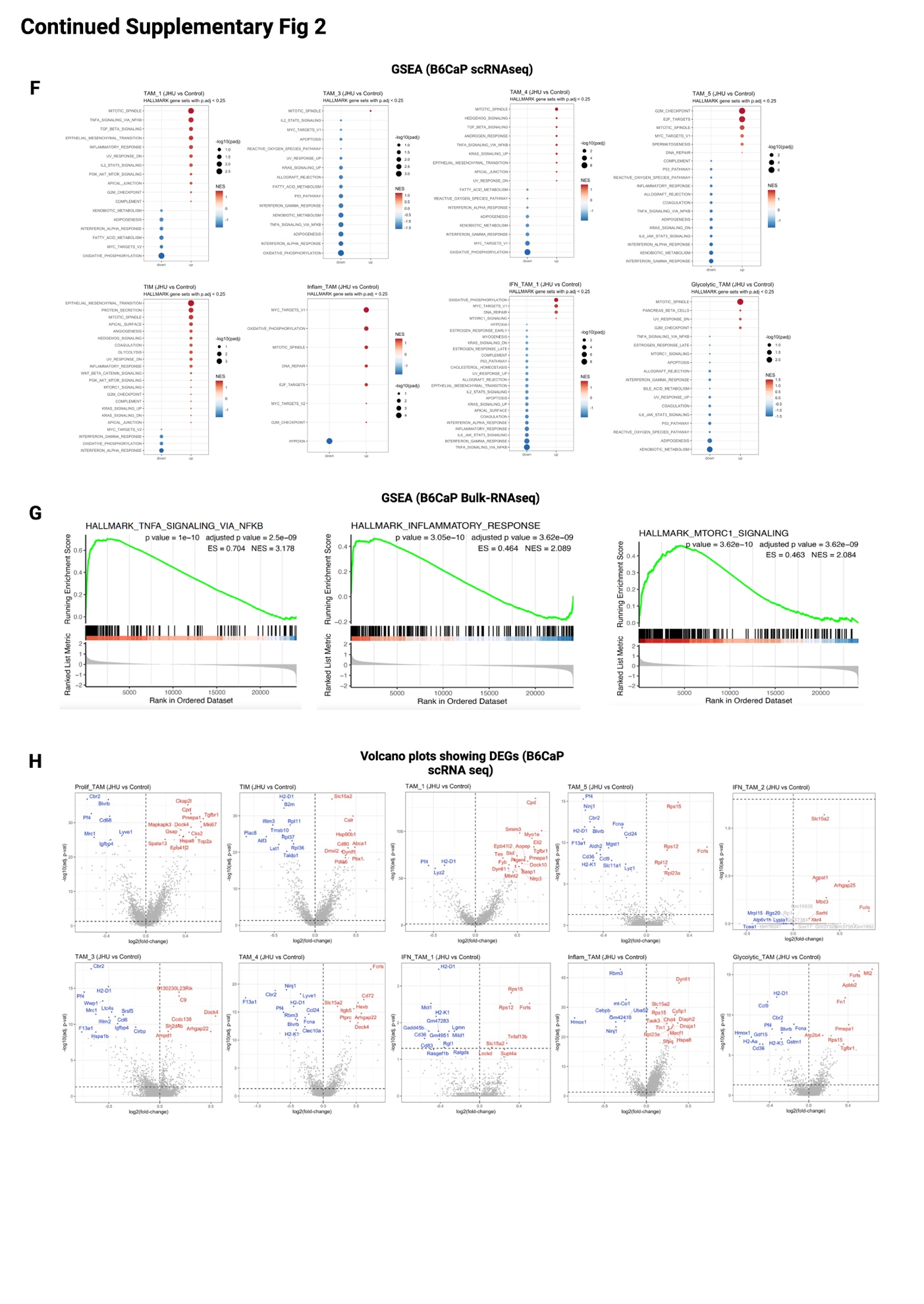
**

**
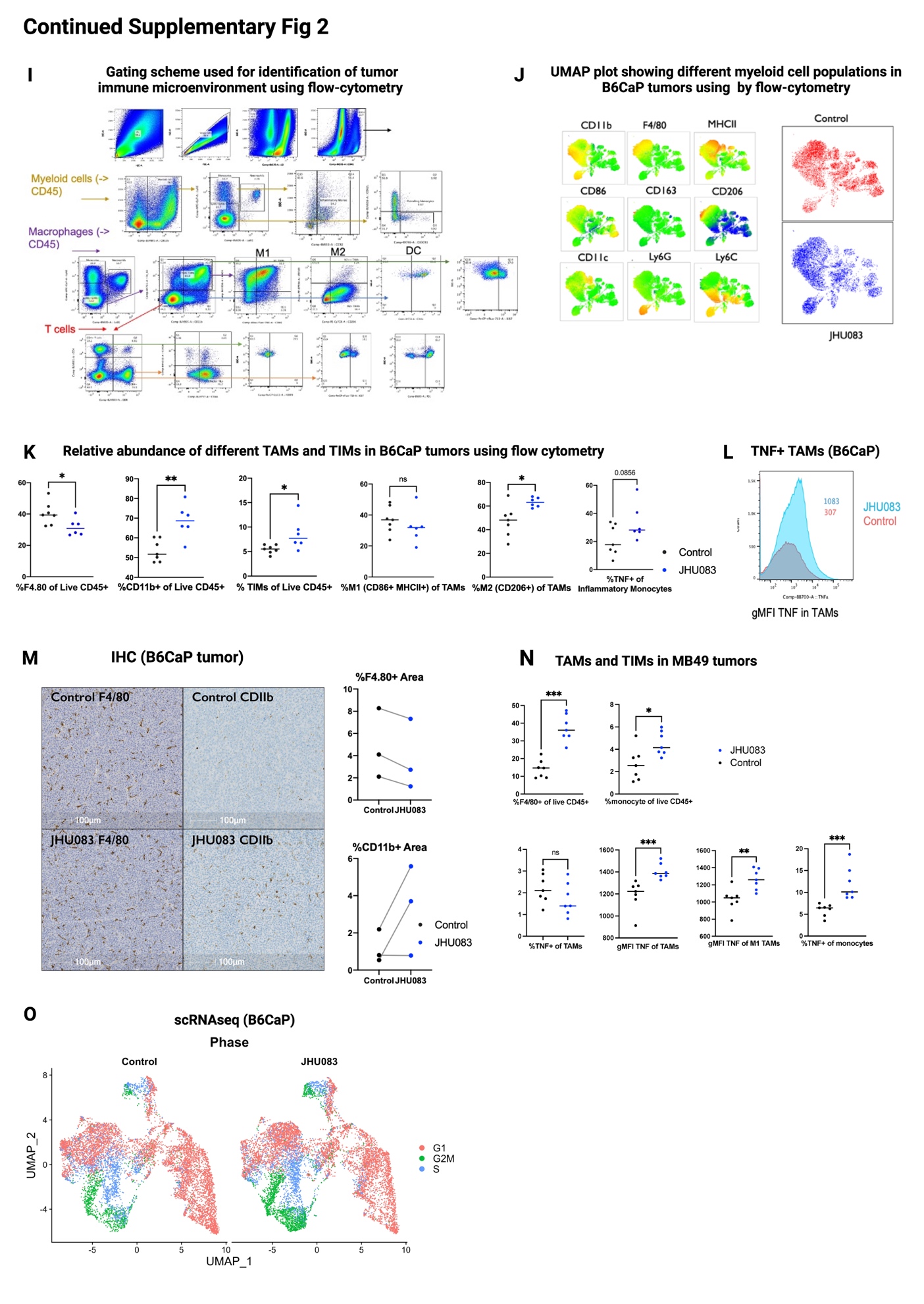
**

**Supplementary Figure 2. (A)** Tumor volume measurement of B6CaP tumors at an early time point following JHU083 treatment. Briefly, control and JHU083-treated tumors were used for scRNAseq (Day 7 post-treatment) (n=6) on enriched live CD45^+^ and CD45^-^ cells. **(B)** Tumor volume measurement of B6CaP tumors following JHU treatment. Tumors at a late time point (Day 18 post-treatment) (n=6/ group) were used for isolation of FACS-sorted TAMs (Live CD45^+^ CD3^-^ Ly6G^-^ CD11b^+^ F4.80^+^) for bulk RNAseq. The diagram on the right-hand side shows the PCA analysis. **(C)** UMAP plots showing different immune cell clusters within the CD45^+^ compartment following scRNAseq (left) and dot plot showing expression levels of marker genes identified by SingleR algorithm to confirm cell-type identities (right) across all samples. **(D)** UMAP plots showing identification and relative abundance of different immune cells within CD45^+^ compartments compared between JHU083-treated cells vs. control samples (left). Density plot (UMAP) showing the differential abundance of JHU083-treated vs. control samples (right). **(E)** Dot plot showing highly expressed marker genes within each TAM/TIM cluster identified within the parent macrophage/monocyte cluster (*Adgre1^+^ Mrc1^+^Itgam^+^Ccr2^+^*). **(F)** Dot plots showing relative enrichment of different hallmark pathways using GSEA (data obtained from bulk RNAseq of TAMs in JHU083-treated vs. control) in each cluster of the macrophage/monocyte subset. **(G)** GSEA enrichment plots for the Hallmark TNFA_signaling_via_NfKb, Inflammatory_response, and mTORC1_signaling gene sets from bulk RNA-sequencing of TAMs (JHU083-treated vs. control). **(H)** Volcano plots representing top DEGs from TAM/TIM clusters identified in scRNAseq (JHU083-treated vs. control). **(I)** Gating schematic utilized for flow cytometry-based analyses of the tumor microenvironment (TME) in MB49 and B6CaP tumors based on cell-surface and intracellular expression of phenotypic markers. **(J)** UMAP plots showing different myeloid cell populations and their relative abundance in B6CaP tumors using flow cytometry analyses in JHU083-treated vs. control tumors. **(K)** Differential abundance of different TAM and TIM subsets in B6CaP tumors (control vs. JHU083-treated) based on cell-surface and intracellular expression of different phenotypic markers using flow cytometry. **(L)** Geometric mean of TNF expression of a representative TAM population in control vs. JHU083-treated tumors. **(M)** Representative immunohistochemistry (IHC) images and quantification of F4/80 and CD11b in three independent JHU083 treated B6CaP tumors experiments (IHC was performed in a single tumor randomly selected from each experiment). **(N)** Differential abundance of TAMs and TIMs, percentage of TNF^+^ TAMs and TIMs, also gMFI of TNF in TAMs in MB49 tumors based on cell-surface and intracellular expression of phenotypic markers using flow cytometry. **(O)** UMAP plots of the macrophage/monocyte subset and their predicted cell cycle phases. DEGs were calculated with DESeq2 in the bulk RNA-seq data, and with the Wilcoxon rank-sum test for the scRNA-seq data. Statistical analyses done with either t-test or two-way ANOVA using Bonferroni's multiple comparisons (**P* < 0.05, ***P* < 0.01, ****P* < 0.001, *****P* < 0.0001).
