# Supplementary material for: Metabolic Reprogramming of Tumor-Associated Macrophages Using Glutamine Antagonist JHU083 Drives Tumor Immunity in Myeloid-Rich Prostate and Bladder Cancers
Source: Cancer Immunol Res. 2024 Apr 26;12(7):854–75. doi: 10.1158/2326-6066.CIR-23-1105 (PMC11217738; doi:10.1158/2326-6066.CIR-23-1105)
Supplement: Supplementary Figure 3 [file cir-23-1105_supplementary_figure_3_suppsf3.docx]

**
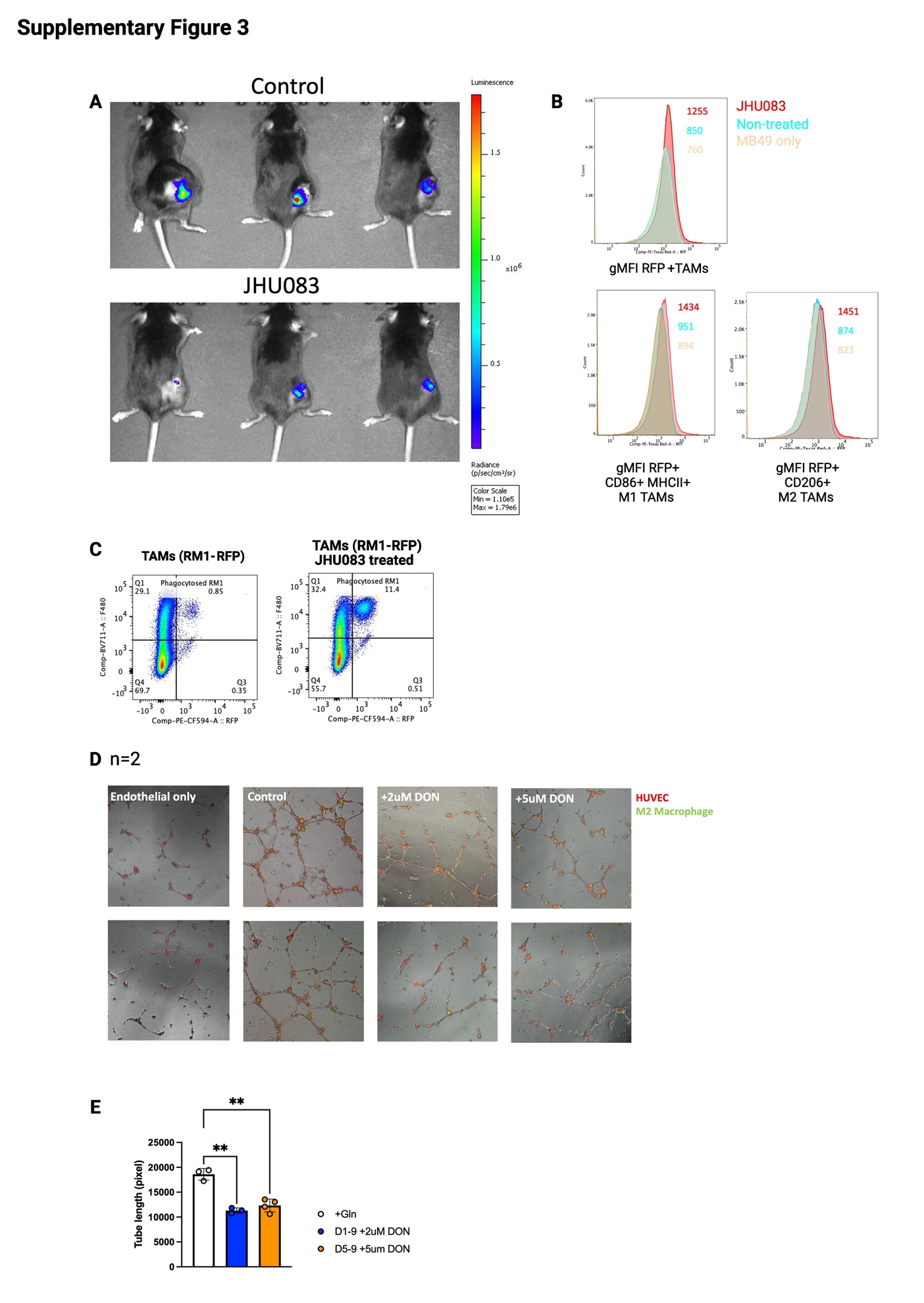
**

**Supplementary Figure 3.** **(A)** IVIS-based quantification of tumor growth by luciferase bioluminescence activity in MB49-RFP^+^ tumors. **(B)** RFP gMFI expression in TAMs, M1 TAMs, and M2 TAMs and **(C)** Representative flow cytometry plots of RFP^+^ RM1 tumor TAMs. (**D & E**) *In vitro* endothelial assay. Briefly, either non treated or DON-treated, PBMC-derived macrophages were co-cultured with untreated endothelial HUVEC2 cells on solidified Matrigel, and endothelial tube length quantified using ImageJ (n=2 experiments).
