# Supplementary material for: Metabolic Reprogramming of Tumor-Associated Macrophages Using Glutamine Antagonist JHU083 Drives Tumor Immunity in Myeloid-Rich Prostate and Bladder Cancers
Source: Cancer Immunol Res. 2024 Apr 26;12(7):854–75. doi: 10.1158/2326-6066.CIR-23-1105 (PMC11217738; doi:10.1158/2326-6066.CIR-23-1105)
Supplement: Supplementary Figure 4 [file cir-23-1105_supplementary_figure_4_suppsf4.docx]

**
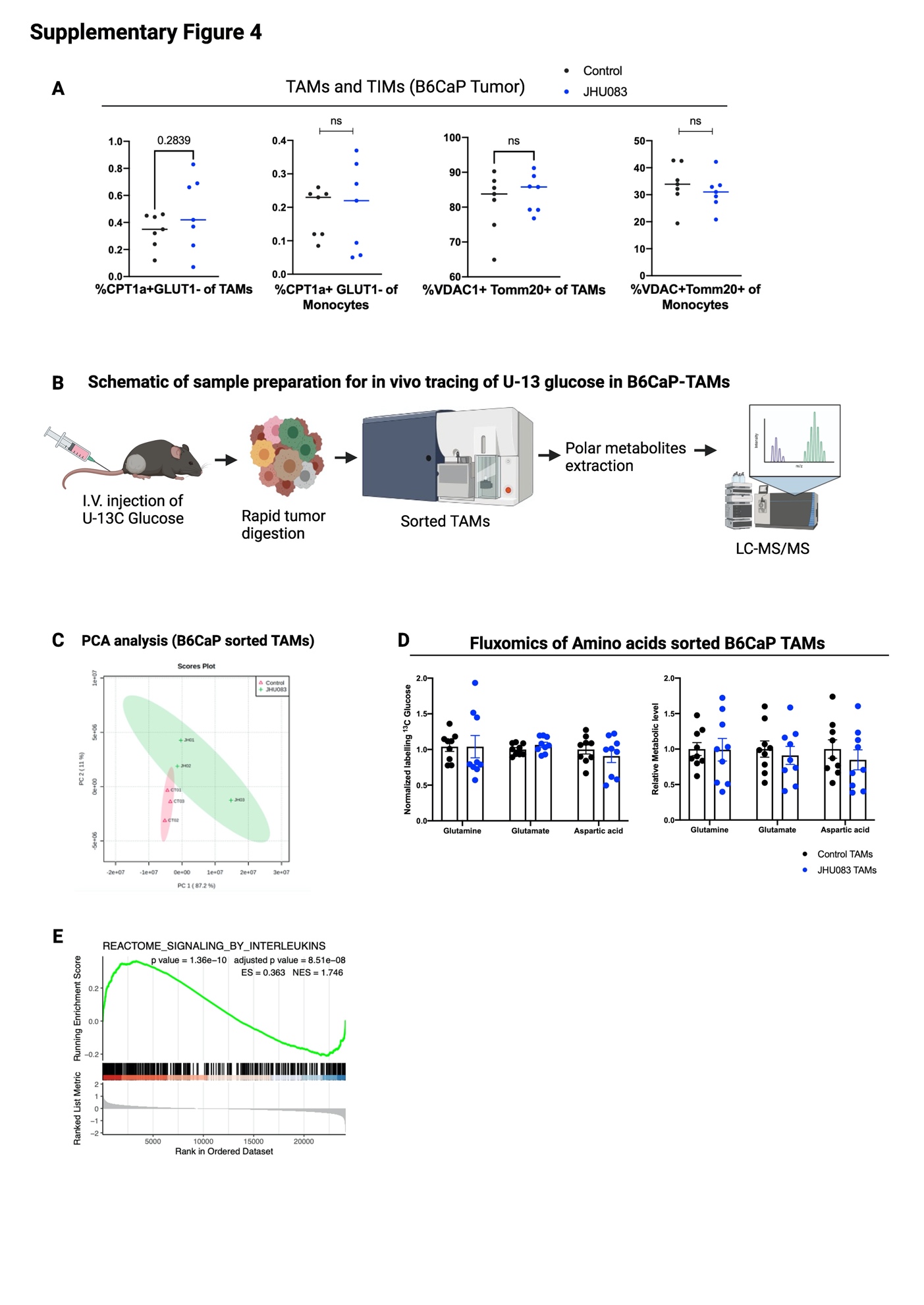
**

**Supplementary Figure 4. Metabolic reprogramming of TAMs after JHU083 treatment.** **(A)** Percentage of CPT1α^+^ GLUT^-^, VDAC1 TOMM20^+^ cells in TAMs and TIMs in B6CaP tumors as determined using flow cytometric analysis **(B)** Schematic diagram showing sample preparation for *in vivo* tracing of U-^13^C glucose in B6CaP TAMs after rapid digestion and FACS sorting. **(C)** PCA analysis of TAMs (B6CaP) for targeted metabolite analysis using LC-MS/MS (n=3/group). **(D)** Normalized relative labeled metabolites and their abundances from U-^13^C glucose in amino acids in TAMs derived from B6CaP tumors (n=9/group from 2 independent experiments), and **(E)** GSEA showing pathway enrichment for the Reactome Signaling by Interleukins from DEGs identified in bulk-RNA seq data in B6CaP sorted TAMs. Statistical analyses were done with unpaired t-test. (**P* < 0.05, ***P* < 0.01, ****P* < 0.001, *****P* < 0.0001).
