# Supplementary material for: Metabolic Reprogramming of Tumor-Associated Macrophages Using Glutamine Antagonist JHU083 Drives Tumor Immunity in Myeloid-Rich Prostate and Bladder Cancers
Source: Cancer Immunol Res. 2024 Apr 26;12(7):854–75. doi: 10.1158/2326-6066.CIR-23-1105 (PMC11217738; doi:10.1158/2326-6066.CIR-23-1105)
Supplement: Supplementary Figure 5 [file cir-23-1105_supplementary_figure_5_suppsf5.docx]

**
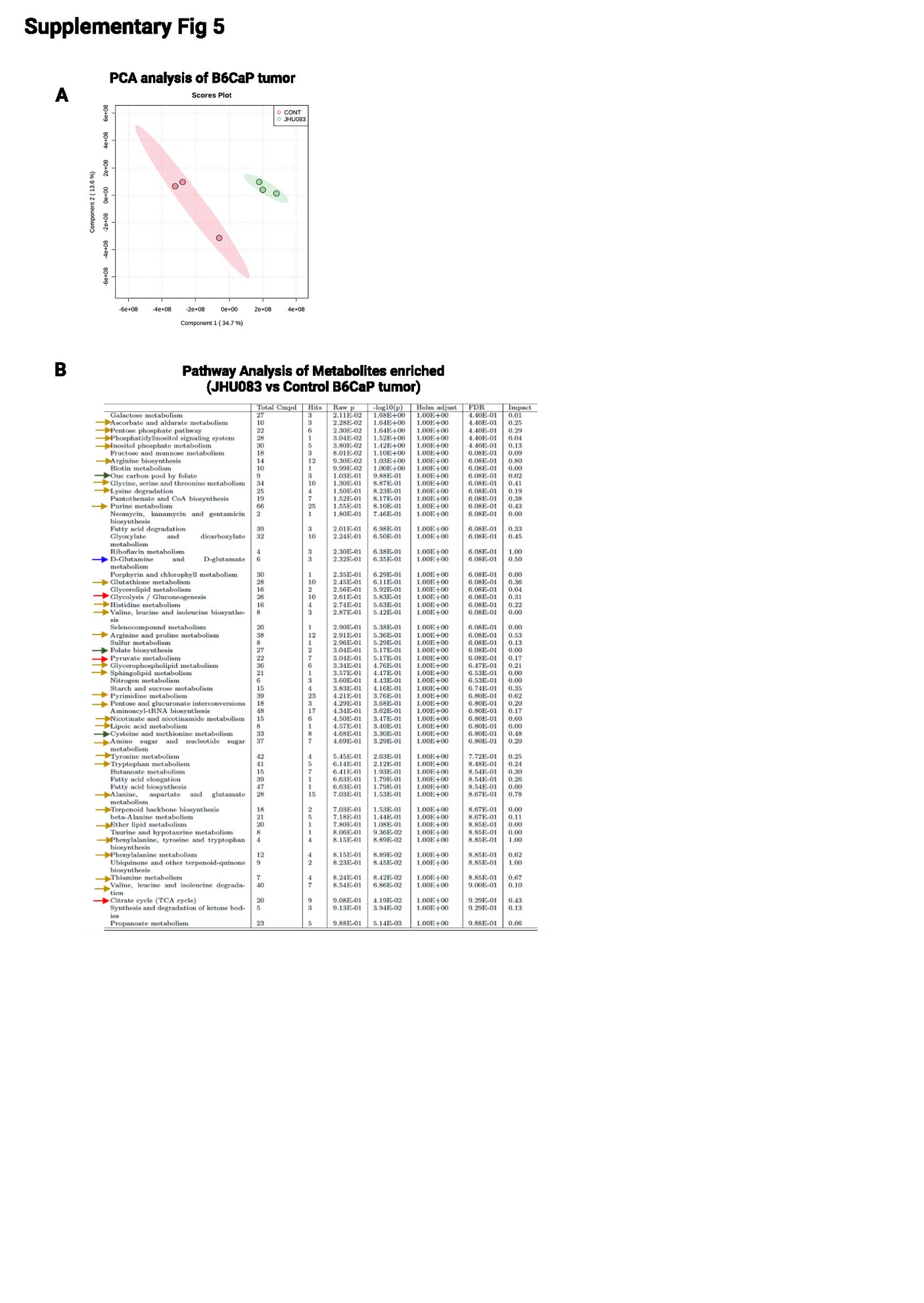
**

**Supplementary Figure 5.** **(A)** PCA analysis of B6CaP tumor samples submitted for LC/MS-MS analysis, and **(B)** Pathway analysis of differential metabolites upregulated by JHU083-treated B6CaP tumors.
