# Supplementary material for: Metabolic Reprogramming of Tumor-Associated Macrophages Using Glutamine Antagonist JHU083 Drives Tumor Immunity in Myeloid-Rich Prostate and Bladder Cancers
Source: Cancer Immunol Res. 2024 Apr 26;12(7):854–75. doi: 10.1158/2326-6066.CIR-23-1105 (PMC11217738; doi:10.1158/2326-6066.CIR-23-1105)
Supplement: Supplementary Figure 6 [file cir-23-1105_supplementary_figure_6_suppsf6.docx]

**
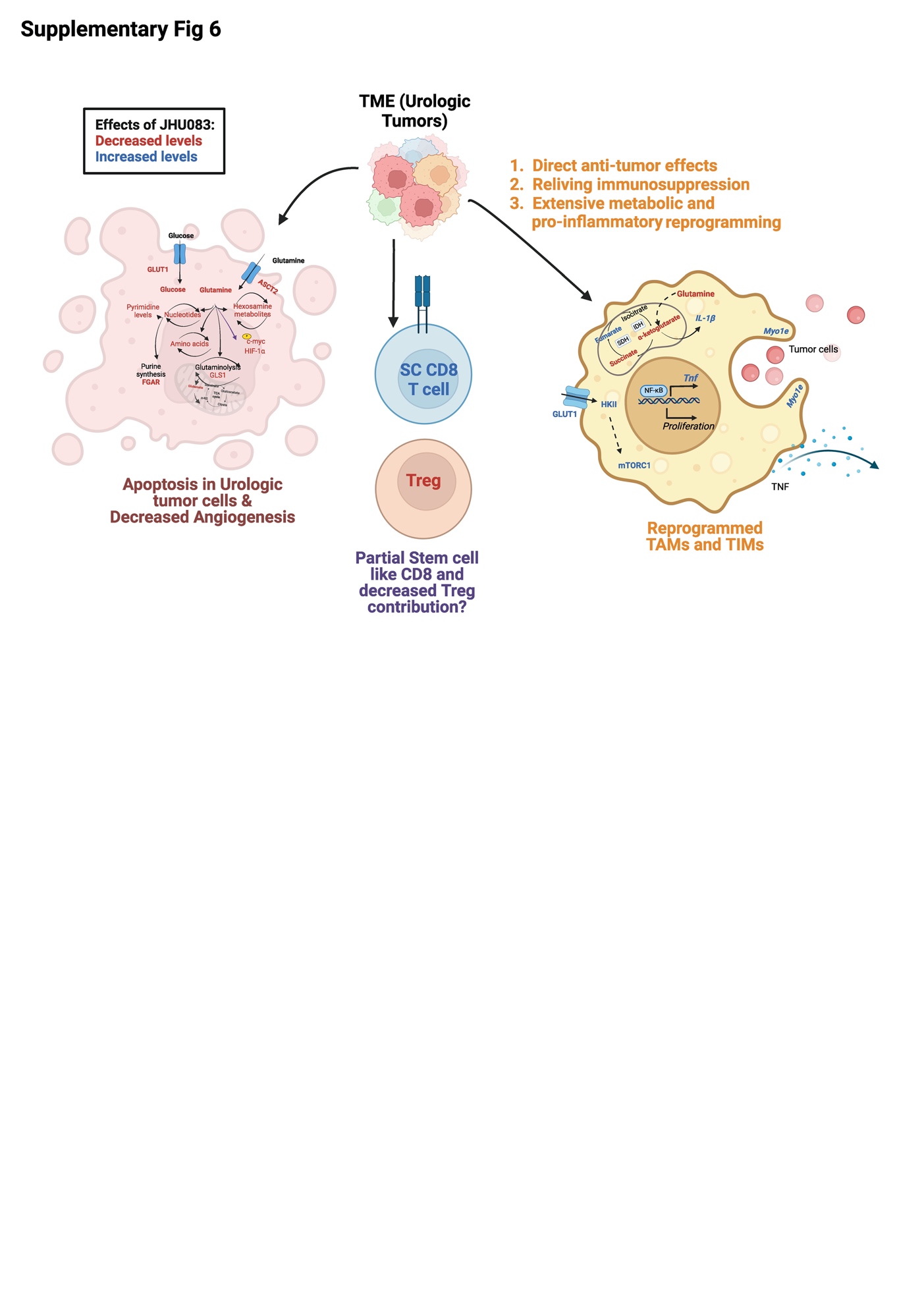
**

**Supplementary Figure 6 (supporting data for figure 6). (A)** Schematic cartoon diagram representation of the proposed model of anti-tumor immunity driven by glutamine inhibition induced by JHU083 in urologic tumors.
